# Non-Enzymatic DNA Cleavage Reaction Induced by 5-Ethynyluracil in Methylamine Aqueous Solution and Application to DNA Concatenation

**DOI:** 10.1371/journal.pone.0092369

**Published:** 2014-03-19

**Authors:** Shuji Ikeda, Kazuki Tainaka, Katsuhiko Matsumoto, Yuta Shinohara, Koji L. Ode, Etsuo A. Susaki, Hiroki R. Ueda

**Affiliations:** 1 Laboratory for Synthetic Biology, Quantitative Biology Center, RIKEN, Chuo-ku, Kobe, Japan; 2 Laboratory for Systems Biology, Center for Developmental Biology, RIKEN, Chuo-ku, Kobe, Japan; 3 Department of Systems Pharmacology, Graduate School of Medicine, The University of Tokyo, Bunkyo-ku, Tokyo, Japan; 4 Department of Biological Sciences, Graduate School of Science, Osaka University, Toyonaka, Osaka, Japan; 5 Graduate School of Frontier Biosciences, Osaka University, Suita, Osaka, Japan; Institute of Molecular Genetics IMG-CNR, Italy

## Abstract

DNA can be concatenated by hybridization of DNA fragments with protruding single-stranded termini. DNA cleavage occurring at a nucleotide containing a DNA base analogue is a useful method to obtain DNA with designed protruding termini. Here, we report a novel non-enzymatic DNA cleavage reaction for DNA concatenation. We found that DNA is cleaved at a nucleotide containing 5-ethynyluracil in a methylamine aqueous solution to generate 5′-phosphorylated DNA fragment as a cleavage product. We demonstrated that the reaction can be applied to DNA concatenation of PCR-amplified DNA fragments. This novel non-enzymatic DNA cleavage reaction is a simple practical approach for DNA concatenation.

## Introduction

Genetic recombination is ubiquitous research tool in the biological sciences. Typically, restriction enzymes are used to cut and paste DNA fragments for genetic recombination [1]. These enzymes cleave double-stranded DNA to produce sticky ends, enabling sequence-specific DNA ligation. The recognition sequences for restriction enzymes, however, impose severe sequence restrictions on the design of recombinant DNA sequences. Recently, various seamless DNA concatenation methods have been reported, in which no specific sequence is required for DNA joining [2]–[10].

Site-specific DNA cleavage using a DNA base analogue can produce DNA with protruding termini which can be used for seamless DNA concatenation [8]–[10]. Giese et al. achieved non-enzymatic nucleotide-specific DNA cleavage by using 8-oxoguanine as a degradable DNA base to produce single-stranded overhangs on PCR-amplified DNA [10]. Such non-enzymatic DNA cleavage reactions have several advantages. 1) Reagents for non-enzymatic reactions are generally less expensive than enzymes. 2) Quality control of the reagents is easier than for enzymes. 3) Non-enzymatic reactions are more often tolerent of pH and ion concentrations than their enzymatic counterparts. Their method does have two major limitations — the mutagenicity of 8-oxoguanine and the requirement of O_2_ bubbling for the degradation of DNA containing 8-oxoguanine.

**Figure 1:**
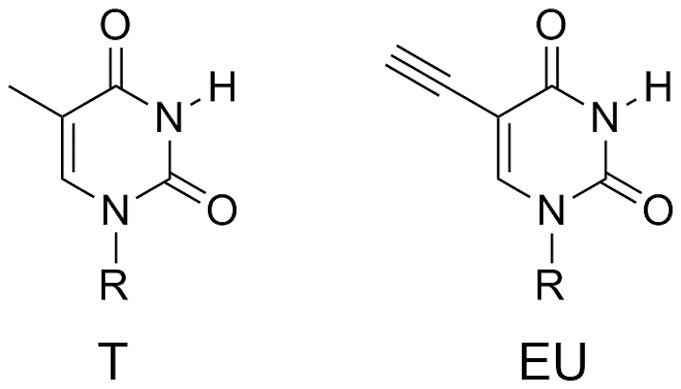
Chemical structures of thymine (T) and 5-ethynyluracil (EU).

Here, we report a novel DNA cleavage reaction induced by 5-ethynyluracil. The reaction occurs in a methylamine aqueous solution to cause DNA cleavage at a nucleotide containing 5-ethynyluracil. One of the cleavage products is a 5′-phosphorylated DNA fragment which is favourable for enzymatic ligation. We applied the reaction to the cleavage of PCR-amplified DNA fragments and showed the resulting DNA fragments can be concatenated. The DNA cleavage requires only the addition and removal of methylamine enabling a simple procedure for DNA concatenation. Sequencing results also indicate that the mutagenicity of 5-ethynyluracil might be low as would be expected given its structural similarity to thymine (Figure 1).

## Materials and Methods

### General

DNA oligonucleotides were synthesized on an NTS H-6 DNA/RNA synthesizer. Analysis and purification of DNA oligonucleotides by reversed-phase HPLC was carried out on a CHEMCOBOND 5-ODS-H column (10 mm×150 mm) with a Gilson Chromatograph, Model 305. Flow rate of the solvent for HPLC was 3.0 mL•min^−1^. Detection wavelength of the UV detector for HPLC analysis was 254 nm. MALDI TOF mass spectra were measured with a Shimadzu AXIMA Assurance. DNA concentration was calculated from UV absorbance at 260 nm [11]. Molar extinction coefficients of DNA oligonucleotides were calculated by regarding 5-ethynyluracil as thymine.

### Cleavage of DNA oligonucleotides

Synthesis of DNA oligonucleotides containing 5-ethynyluracil is described in the Supporting Information (Method S1) [12], [13]. The same volume of 28% NH_3_aq or 40% MeNH_2_aq was added to the aqueous solution of DNA oligonucleotides (100 μM) in a screw-cap tube for the DNA cleavage. After incubation at 70, 37, or 25 °C, methylamine in the reaction solutions was removed by speed-vac. The residue was directly analysed by reversed-phase HPLC with a linear gradient over 20 minutes from 5 to 20% CH_3_CN in 50 mM ammonium formate (AF). The cleavage products were purified by reversed-phase HPLC, desalted and identified by MALDI TOF mass spectrometry (Data S1).

### DNA concatenation

PCR amplification of DNA fragments using primers containing 5-ethynyluracil is described in the Supporting Information (Method S2) [12], [13]. The PCR sample was transferred from the PCR tube to a screw-cap tube. The same volume of 40% MeNH_2_aq was added to the PCR sample to cleave DNA. The solutions were incubated at 25°C for 48 hours, 37°C for 10 hours, and 70°C for 0.5 hours, respectively. Methylamine in the sample was removed by speed-vac. H_2_O was added to return the sample to its post-PCR volume. The solutions of 1.5 and 2.2 kbp DNA fragments were mixed in a 1:1 ratio and incubated at 40°C for 10 minutes for hybridization. The mixed solution was diluted 20× with H_2_O for the transformation of competent cells (TOYOBO, Competent high DH5α). 1 μL of the diluted solution and 10 μL of thawed competent cells were added to an ice-cold tube and the mixture was left on ice for 30 minutes. After heat-shock at 42°C for 45 seconds, SOC medium (200 μL) was added to the mixture. The mixture was incubated at 37°C for 30 minutes and 50 μL of the mixture was plated on four LB agar plates containing ampicillin (50 μg•mL^−1^). After incubation at 37°C for 16 hours, the numbers of the transformants on the plates were counted. The transformants were picked up from the colonies and cultured in 3 mL LB medium containing ampicillin (50 μg•mL^−1^) at 37°C for 16 hours. Plasmid was purified from the culture by using the Wizard Plus minipreps DNA purification system (Promega). Sequencing of the plasmids was carried out by using BigDye Terminator v3.1 Cycle Sequencing Kit (Applied Biosystems) and an ABI3130 or 3170 automated sequencer (Applied Biosystems).

## Results and Discussion

**Figure 2:**
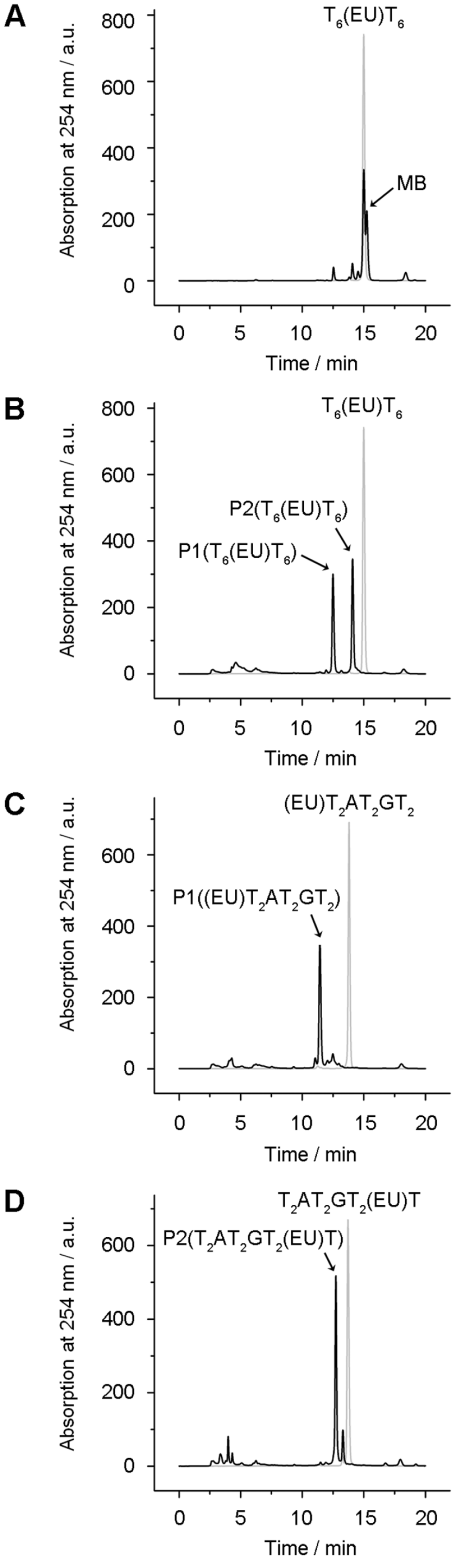
Degradation of DNA oligonucleotides containing 5-ethynyluracil. (A), (B) HPLC charts of T_6_(EU)T_6_ before (gray) and after (black) the reaction in 14% NH_3_aq (A) or 20% MeNH_2_aq (B) at 70°C for 2 hours. (C), (D) (EU)T_2_AT_2_GT_2_ (C) and T_2_AT_2_GT_2_(EU)T (D) before (gray) and after (black) the reaction in 20% MeNH_2_aq at 70°C for 2 hours.

The base induced-cleavage reaction of DNA containing 5-ethynyluracil has not previously been described in detail. Seela et al reported that DNA oligonucleotides containing 5-ethynyluracil are unstable at 55°C in aqueous ammonia, generating by-products [12]. In order to investigate the degradation reaction, we prepared DNA oligonucleotides containing 5-ethynyluracil (EU) according to the literature [12], [13]. As reported, a DNA oligonucleotide, T_6_(EU)T_6_, generated the by-products under heating in aqueous ammonia (Figure 2A). MALDI -TOF mass data indicate that the main peak in Figure 2A corresponds to T_6_(EU)T_6_. The molecular mass of the main by-product (MB), appearing just after the main peak, is larger by 17 than that of T_6_(EU)T_6_.This indicates that MB might be generated by addition of one molecule of ammonia to T_6_(EU)T_6_.

To our surprise, we found that methylamine, which is a stronger nucleophile than ammonia, caused nearly quantitative DNA cleavage of T_6_(EU)T_6_ at the nucleotide containing 5-ethynyluracil. Two products, P1(T_6_(EU)T_6_) and P2(T_6_(EU)T_6_), appeared as two peaks on the reversed-phase HPLC chart after the reaction (Figure 2B). The molecular masses of P1(T_6_(EU)T_6_) and P2(T_6_(EU)T_6_) are indicated as 1843 and 1972 by MALDI TOF mass spectrometry (Data S1). The results indicate that T_6_(EU)T_6_ was cleaved at the EU nucleotide. P1(T_6_(EU)T_6_) was expected to be either pT6 or T6p because the molecular mass is identical to those of pT6 and T6p. P2(T_6_(EU)T_6_) likely contains a residue derived from the EU nucleotide because the molecular mass is larger than those of pT6 and T6p. Most of the T_6_(EU)T_6_ was cleaved after 30 minutes at 70°C, 10 hours at 37°C, and 48 hours at 25°C in 20% MeNH_2_aq (Figure S1).

**Figure 3:**
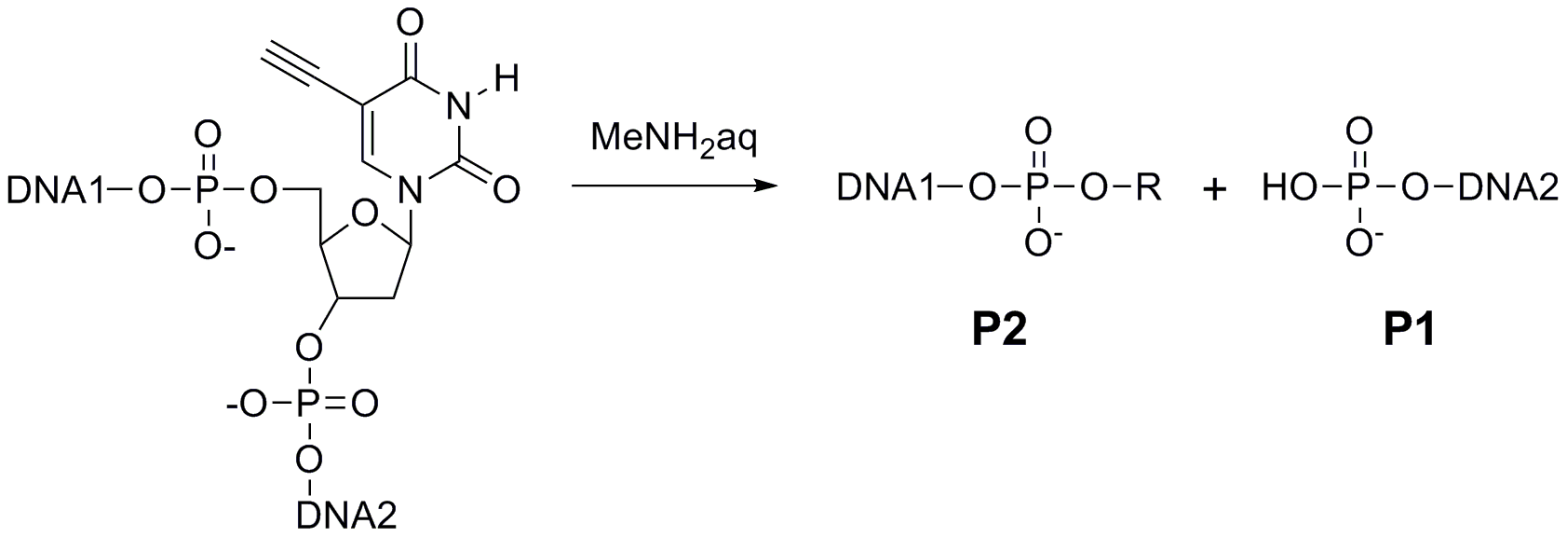
Chemical formula of the DNA cleavage reaction. R is expected to be an abasic sugar derivative.

The cleavage reaction was examined in detail by using (EU)T_2_AT_2_GT_2_ and T_2_AT_2_GT_2_(EU)T. Treatment of (EU)T_2_AT_2_GT_2_ with methylamine generated a single main product, namely, P1((EU)T_2_AT_2_GT_2_) (Figure 2C). P1((EU)T_2_AT_2_GT_2_) was identified as pT_2_AT_2_GT_2_ by MALDI TOF mass spectrometry (Data S1) and coinjection with authentic pT_2_AT_2_GT_2_ [14] to reversed-phase HPLC. The methylamine treatment with T_2_AT_2_GT_2_(EU)T generated a single main product, namely, P2(T_2_AT_2_GT_2_(EU)T) (Figure 2D). The molecular mass of P2(T_2_AT_2_GT_2_(EU)T) was larger by 129 than that of T_2_AT_2_GT_2_p (2486) (Data S1). Heating at 120°C in a buffer degraded P2(T_2_AT_2_GT_2_(EU)T) to produce a major product, namely, P2' (T_2_AT_2_GT_2_(EU)T) (Figure S2). The molecular mass of P2' (T_2_AT_2_GT_2_(EU)T) was indicated as 2486 by MALDI TOF mass spectrometry (Data S1). Dephosphorylation of P2' (T_2_AT_2_GT_2_(EU)T) by alkaline phosphatase generated T_2_AT_2_GT_2_, which was confirmed by MALDI TOF mass spectrometry (Data S1) and coinjection to reversed-phase HPLC with the authentic T_2_AT_2_GT_2_. The results strongly suggest that P2' (T_2_AT_2_GT_2_(EU)T) is T_2_AT_2_GT_2_p. It has been reported that 3'-phosphorylated DNA fragments can be generated by heat degradation of DNA containing an abasic site [15], suggesting that the structure of P2(T_2_AT_2_GT_2_(EU)T)) might be similar to a cleavage product obtained by elimination of the DNA base [16]. We show the chemical formula of the DNA cleavage reaction in Figure 3.

DNA oligonucleotides, T_5_X(EU)XT_5_ (X  =  A, C, and G) and CGCA_2_T(EU)TA_2_CGC, were also cleaved to produce two products corresponding to P1 and P2 (Figure S3). Here, we used volatile methylamine to facilitate removal from the samples. Other primary amines such as ethylenediamine and 2-aminoethanol can be also used for the DNA cleavage reaction (data not shown). We have named the DNA cleavage reaction QBIC (Quantitative Base-Induced DNA Cleavage), as the reaction proceeded almost quantitatively. We propose a cyclization-driven base elimination induced by nucleophilic attack by methylamine as the mechanism of the DNA cleavage reaction induced by 5-ethynyluracil (Method S3). The DNA cleavage by other DNA base analogues is consistent with the mechanism (Method S3, Figure S4) [17]–[26]. However, the rate of the DNA cleavage induced by the DNA analogues was slower than that using EU. Our preliminary results indicate that the reaction might be applicable to the preparation of 5′-phosphorylated DNA oligonucleotides by using the DMTr-ON method on a DNA synthesizer (Method S4, Figure S5) [17].

**Figure 4:**
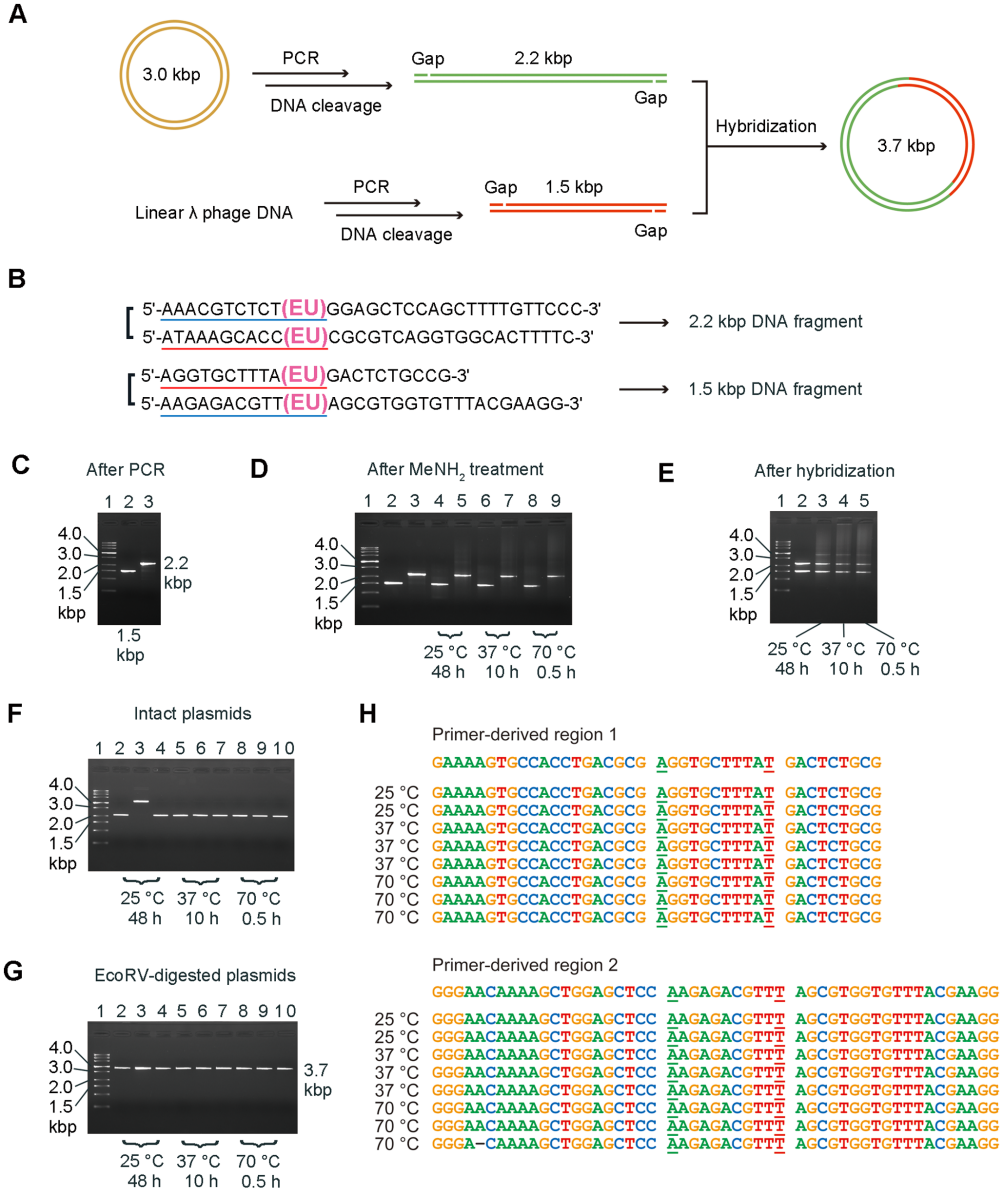
Construction of plasmid from two PCR-amplified DNA fragments. (A) Scheme of plasmid construction. (B) Primer sequences used for PCR. The two sequences underlined in red and blue are complementary to each other. (C–G) Pictures of agarose gel electrophoresis. (C) PCR-amplified DNA fragments 1.5 (lane 2) and 2.2 kbp (lane 3). (D) 1.5 and 2.2 kbp DNA fragments before (lane 2,3) and after DNA cleavage at 25°C for 48 h (lane 4,5), 37°C for 10 h (lane 6,7), and 70°C for 0.5 h (lane 8,9). MeNH_2_ was removed from the samples by speed-vac before electrophoresis. (E) Hybridized 1.5 and 2.2 kbp DNA fragments derived from those without cleavage reaction (lane 2) and cleaved at 25°C for 48 h (lane 3), 37°C for 10 h (lane 4), and 70°C for 0.5 h (lane 5). (F,G) Intact purified plasmids (F) and EcoRV-digested plasmids (G) derived from the DNA fragments cleaved at 25°C for 48 h (lane 2,3), 37°C for 10 h (lane 4–6), and 70°C for 0.5 h (lane 7–9). (H) Sequencing results of primer-derived regions of the plasmids. Underlined letters correspond to EU in the primers.

We applied the QBIC reaction to the concatenation of PCR-amplified DNA fragments. PCR amplification of a DNA fragment with primers containing 5-ethynyluracil generates DNA fragments containing 5-ethynyluracil in the primer-derived regions. Cleavage of the PCR-amplified DNA fragment would produce a gap. If the terminal regions of the two DNA fragments are complementary to each other, they should be able to hybridize by heating and cooling. To verify this, a simple plasmid construction was carried out (Figure 4A). Before PCR, a DNA oligonucleotide containing A, C, G, T, and EU bases was confirmed to be stable under PCR conditions (Figure S6).

Two pairs of primers shown in Figure 4B were used to amplify 1.5 and 2.2 kbp DNA fragments from linear lambda phage DNA and EcoRI-digested pBluescript-sk(–) [27], respectively. Successful amplification of the fragments was indicated by agarose gel electrophoresis (Figure 4C). The same volume of 40% methylamine aqueous solution was added to the PCR samples and the mixtures were incubated at 70°C for 30 minutes, 37°C for 10 hours, and 25°C for 48 hours, respectively. After the cleavage reaction, methylamine in the samples was removed by speed-vac and the sample was diluted with H_2_O to restore the volume it had after PCR. The cleaved DNA fragments were analyzed by agarose gel electrophoresis (Figure 4D). The bands corresponding to the1.5 and 2.2 kbp DNA fragments were slightly weaker after cleavage at higher temperature possibly due to alkali denaturation.of the DNA fragments. The same volume solutions of the cleaved DNA fragments were mixed and the resultant solution was incubated at 40°C for 10 minutes and cooled to 25°C for hybridization. The hybridized samples were analyzed by agarose gel electrophoresis (Figure 4E). Weak bands with a size larger than those of the two DNA fragments indicated that at least a part of them hybridized to form longer DNA fragments, although it was not clear that all the hybridized structures of the two DNA fragments were stable throughout the agarose gel electrophoresis.

The hybridized samples were diluted 20 times with H_2_O. *Escherichia coli* (DH5α) was transformed using 1 μL of the diluted samples. The transformants were plated on LB plates containing ampicillin. The numbers of the transformed DH5α colonies were 59±8, 53±17, and 17±2 for samples with DNA cleavage temperatures of 25°C, 37°C, and 70°C, respectively. Three plasmids were purified from the transformants derived from each DNA sample. The agarose gel electrophoresis of the intact plasmids (Figure 4F) indicated that the size of one plasmid at lane 3 was larger than those of the other plasmids. Cleavage of the plasmids by EcoRV, which would cut one site of the 1.5 kbp fragment, produced 3.7 kbp fragments (Figure 4G) as expected. Those results indicated that the larger plasmid was the dimer of the 3.7 kbp DNA fragment. Sequencing of the primer-derived region of the eight monomer plasmids made it clear that 5-ethynyluracil was correctly replicated as T in the concatenated DNA (Figure 4H). DNA concatenation experiments were carried out for 14 other clones as well; mutations caused by 5-ethynyluracilwere never observed (data not shown). One deletion of the plasmid derived from the sample cleaved at 70°C was presumably caused by insufficient deprotection of the DMTr group during the automated DNA synthesis of the primer.

## Conclusions

In this study, we report a novel DNA cleavage reaction occurring at a nucleotide containing 5-ethynyluracil in a methylamine aqueous solution. Although the reaction rate is faster at elevated temperatures, the reaction proceeded even at room temperature. One cleavage product is a 5′-phosphorylated DNA fragment, which is favourable for applications using enzymatic DNA ligation. We applied the reaction to cleave PCR-amplified DNA fragments, hybridized the DNA fragments, and showed that concatenation of the DNA fragments can be achieved in *Escherichia coli*. The sequencing data of the concatenated DNA indicats high fidelity of 5-ethynyluracil as a template for DNA replication. Because DNA cleavage requires only addition and removal of methylamine, this procedure for DNA concatenation is quite simple.

## Supporting Information

Figure S1 **DNA cleavage of T_6_(EU)T_6_ at 25, 37, and 70°C in 20% MeNH_2_aq.** (A–C), HPLC charts of T_6_(EU)T_6_ before (gray) and after (black) the reaction in 20% MeNH_2_aq at 70°C for 0.5 hours (A), 37°C for 10 hours (B), and 25°C for 48 hours (C).
(TIF)

Figure S2 **Heat degradation of PB'.** HPLC charts of P2(T_2_AT_2_GT_2_(EU)T) before (gray) and after (black) the reaction in sodium phosphate buffer (100 mM, pH 7.0 at 25°C) at 120°C for 2 hours.
(TIF)

Figure S3 **Reactivity of EU in various sequences of DNA oligonucleotides.** HPLC charts of T_5_A(EU)AT_5_ (A), T_5_C(EU)CT_5_ (B), T_5_G(EU)GT_5_ (C), and CGCA_2_T(EU)TA_2_CGC (D) before (gray) and after (black) reaction in 20% MeNH_2_aq at 70°C for 2 hours.
(TIF)

Figure S4 **Reactivity of DNA oligonucleotides containing designed DNA base analogues.** (A–F) HPLC charts of T_6_(EU)T_6_ (A), T_6_(T1)T_6_ (B), T_6_(C1)T_6_ (C), T_6_(C2)T_6_ (D), T_6_(A1)T_6_ (E), and T_6_(G1)T_6_ (F) before (gray) and after (black) the reaction in 20% MeNH_2_aq at 70°C for 12 hours.
(TIF)

Figure S5 **HPLC analysis of 5′-phosphorylation of DNA oligonucleotide.** HPLC charts of crude DNA solution of DMTr-(PU)T_2_AT_2_GT_2_ after NH_3_ treatment (gray) and crude DNA solution of pT_2_AT_2_GT_2_ after ethylenediamine treatment (black). PU depicts 5-phenylethynyluracil.
(TIF)

Figure S6 **Stability of DNA oligonucleotide containing A, T, G, C, and EU under PCR condition.** HPLC chart of CGCA_2_T(EU)TA_2_CGC in PCR buffer (×1) after a temperature program, 94°C, 2 min → [98°C, 30 sec → 60°C, 30 sec → 68°C, 90 sec] ×30 → 4°C.
(TIF)

Data S1 **MALDI TOF mass data of cleavage products.**
(PDF)

Method S1 **Preparation of DNA oligonucleotides containing 5-ethynyluracil (EU).**
(PDF)

Method S2 **PCR amplification by using primers containing 5-ethynyluracil (EU).**
(PDF)

Method S3 **Preparation and cleavage of DNA oligonucleotides containing DNA base analogues.**
(PDF)

Method S4 **5′-phosphorylation of the DNA oligonucleotide using 5-phenylethynyluracil (PU).**
(PDF)
