# Supplementary figures and images for: Non-Enzymatic DNA Cleavage Reaction Induced by 5-Ethynyluracil in Methylamine Aqueous Solution and Application to DNA Concatenation
Source: PLoS One. 2014 Mar 19;9(3):e92369. doi: 10.1371/journal.pone.0092369 (PMC3960239; doi:10.1371/journal.pone.0092369)

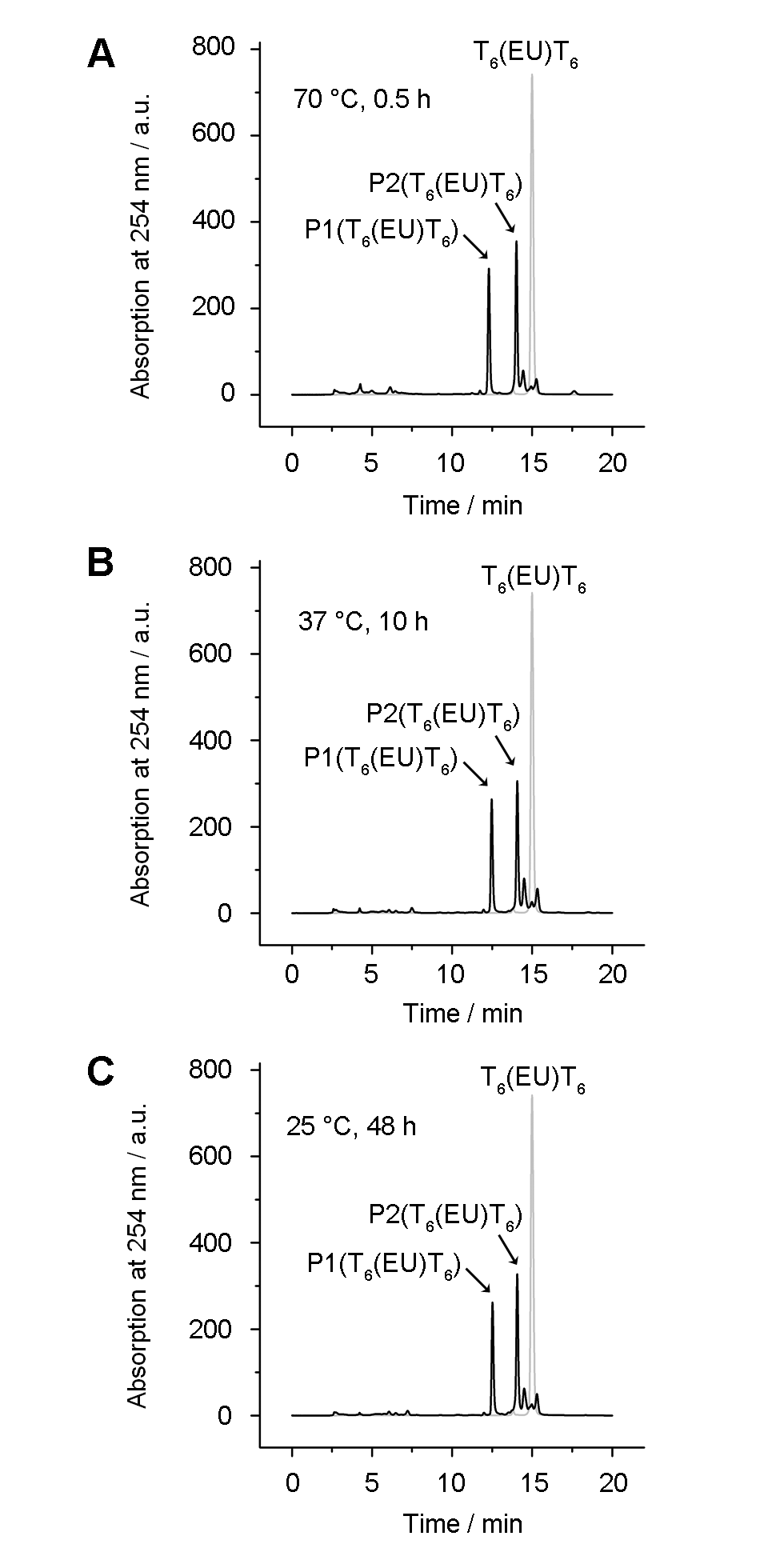

Supplement: Figure S1 — DNA cleavage of T6(EU)T6 at 25, 37, and 70°C in 20% MeNH2aq. (A–C), HPLC charts of T6(EU)T6 before (gray) and after (black) the reaction in 20% MeNH2aq at 70°C for 0.5 hours (A), 37°C for 10 hours (B), and 25°C for 48 hours (C). (TIF) [file pone.0092369.s001.tif]

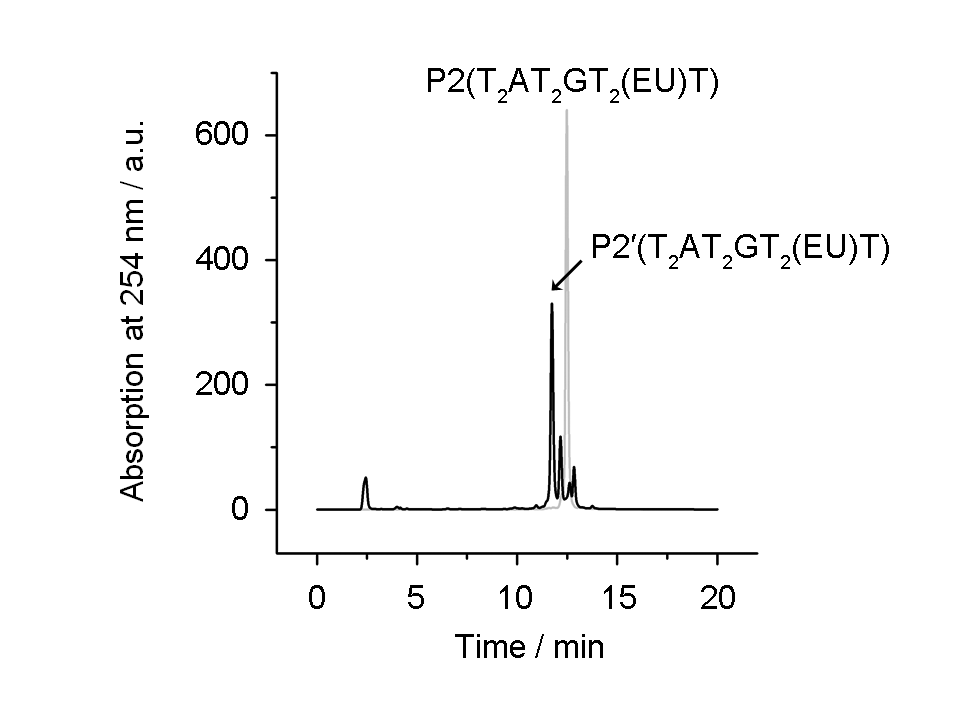

Supplement: Figure S2 — Heat degradation of PB'. HPLC charts of P2(T2AT2GT2(EU)T) before (gray) and after (black) the reaction in sodium phosphate buffer (100 mM, pH 7.0 at 25°C) at 120°C for 2 hours. (TIF) [file pone.0092369.s002.tif]

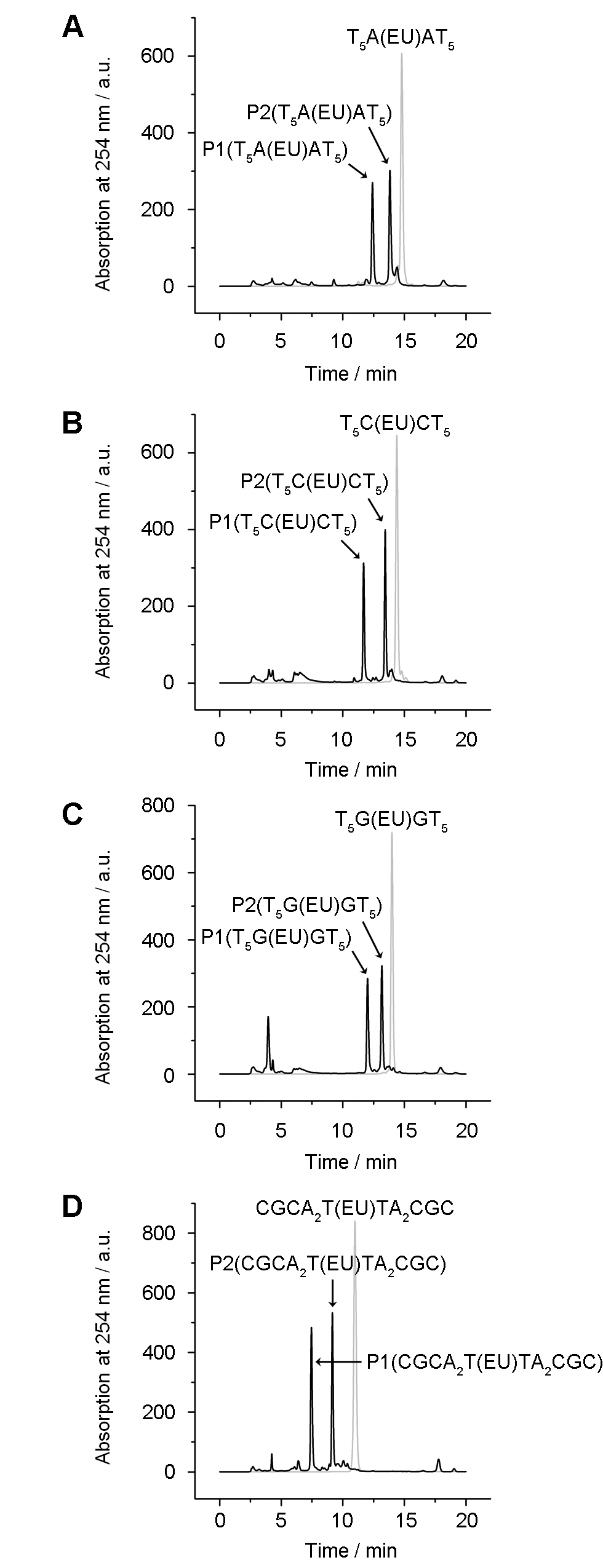

Supplement: Figure S3 — Reactivity of EU in various sequences of DNA oligonucleotides. HPLC charts of T5A(EU)AT5 (A), T5C(EU)CT5 (B), T5G(EU)GT5 (C), and CGCA2T(EU)TA2CGC (D) before (gray) and after (black) reaction in 20% MeNH2aq at 70°C for 2 hours. (TIF) [file pone.0092369.s003.tif]

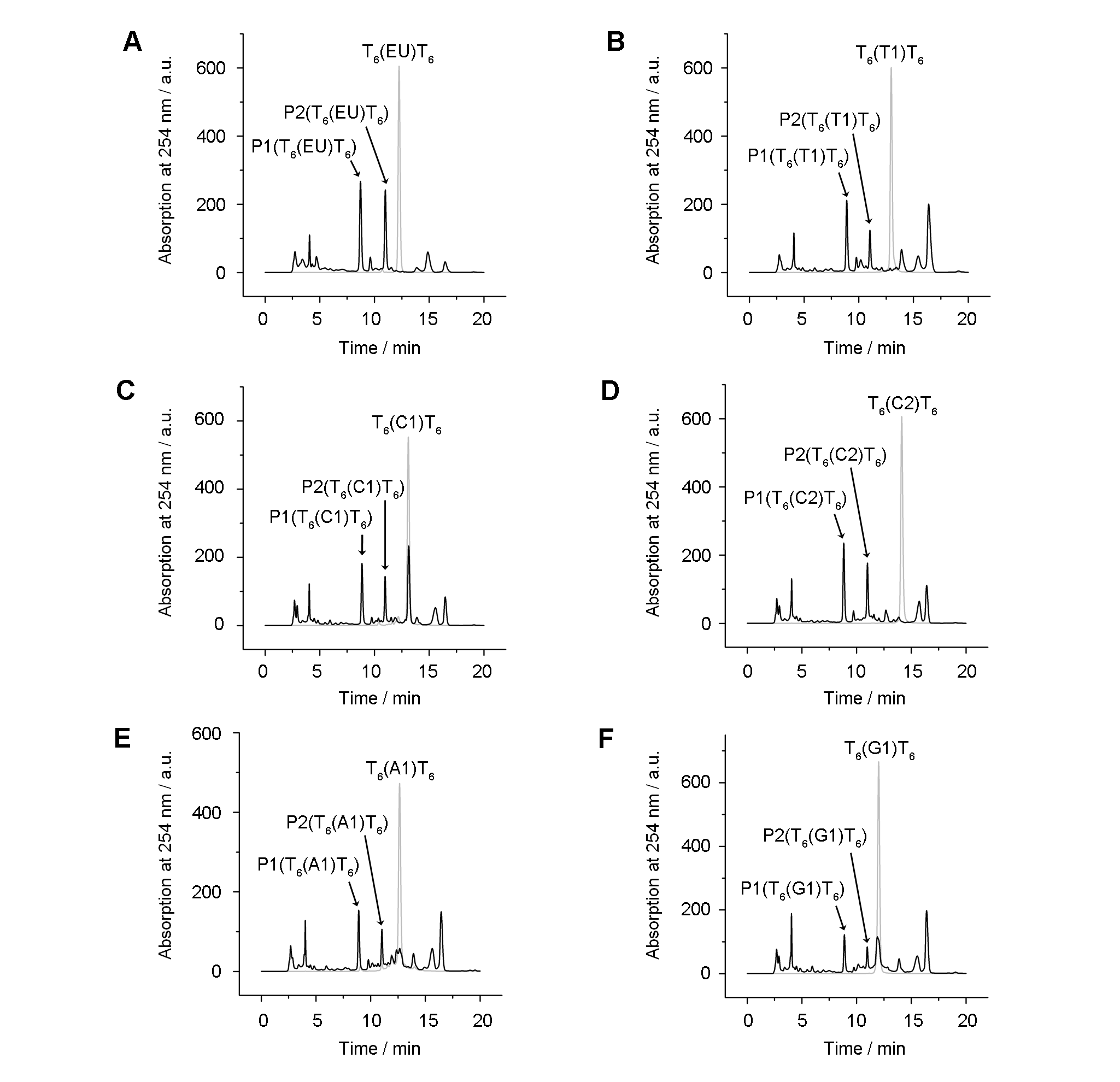

Supplement: Figure S4 — Reactivity of DNA oligonucleotides containing designed DNA base analogues. (A–F) HPLC charts of T6(EU)T6 (A), T6(T1)T6 (B), T6(C1)T6 (C), T6(C2)T6 (D), T6(A1)T6 (E), and T6(G1)T6 (F) before (gray) and after (black) the reaction in 20% MeNH2aq at 70°C for 12 hours. (TIF) [file pone.0092369.s004.tif]

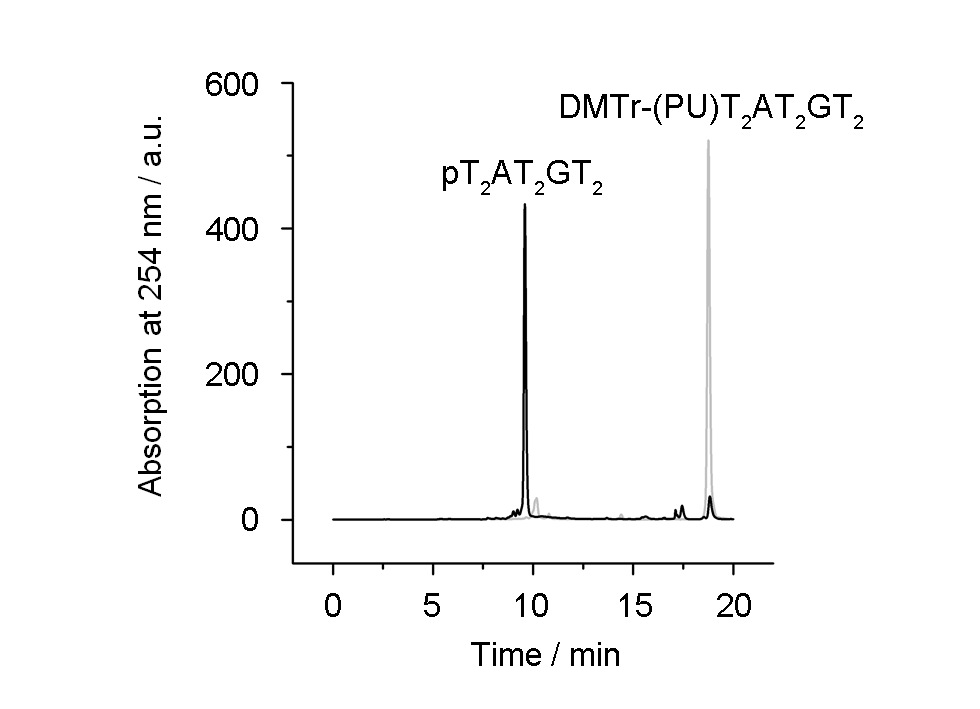

Supplement: Figure S5 — HPLC analysis of 5′-phosphorylation of DNA oligonucleotide. HPLC charts of crude DNA solution of DMTr-(PU)T2AT2GT2 after NH3 treatment (gray) and crude DNA solution of pT2AT2GT2 after ethylenediamine treatment (black). PU depicts 5-phenylethynyluracil. (TIF) [file pone.0092369.s005.tif]

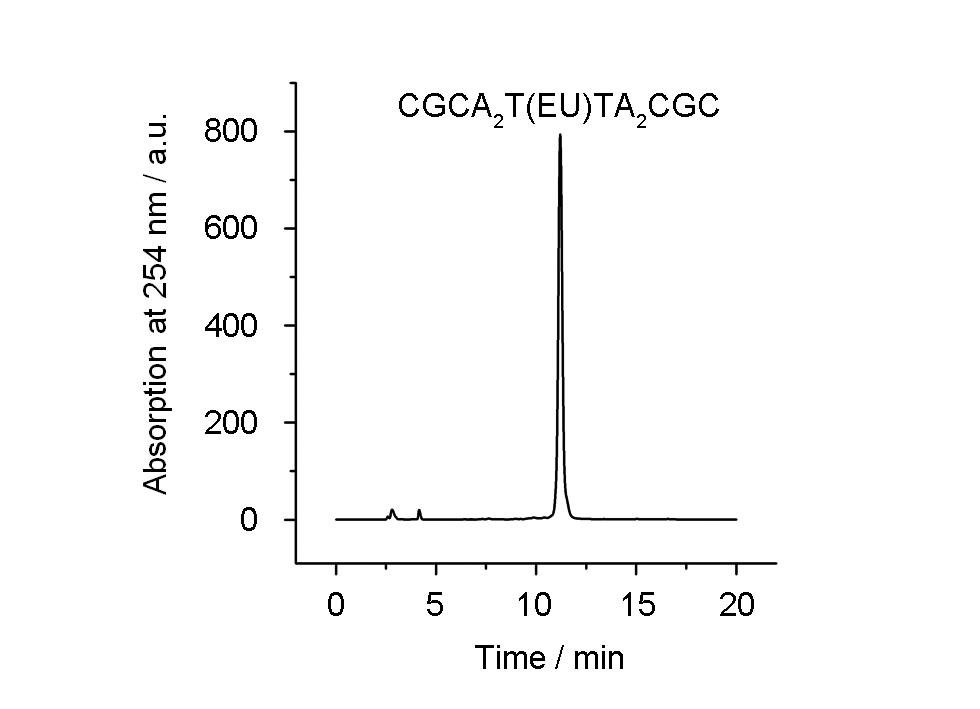

Supplement: Figure S6 — Stability of DNA oligonucleotide containing A, T, G, C, and EU under PCR condition. HPLC chart of CGCA2T(EU)TA2CGC in PCR buffer (×1) after a temperature program, 94°C, 2 min → [98°C, 30 sec → 60°C, 30 sec → 68°C, 90 sec] ×30 → 4°C. (TIF) [file pone.0092369.s006.tif]
