# Supplementary material for: Non-Enzymatic DNA Cleavage Reaction Induced by 5-Ethynyluracil in Methylamine Aqueous Solution and Application to DNA Concatenation
Source: PLoS One. 2014 Mar 19;9(3):e92369. doi: 10.1371/journal.pone.0092369 (PMC3960239; doi:10.1371/journal.pone.0092369)
Supplement: Data S1 — MALDI TOF mass data of cleavage products. (PDF) [file pone.0092369.s007.pdf]

Data S1

MALDI TOF mass data of cleavage products

Products from T_6(EU)T_6; pT_6 (P1), calcd $C_{60}H_{79}N_{12}O_{43}P_6$ 1842.2 [M−H]$^-$; found 1841.8; T_6pR (P2), calcd $C_{66}H_{90}N_{13}O_{45}P_6$ 1971.3 [M−H]$^-$; found 1971.3; Product from (EU)T_2AT_2GT_2; pT_2AT_2GT_2 (P1), calcd for $C_{80}H_{103}N_{22}O_{54}P_8$ 2484.6 [M−H]$^-$; found 2484.6; Product from T_2AT_2GT_2(EU)T; T_2AT_2GT_2pR (P2), calcd for $C_{86}H_{114}N_{23}O_{56}P_8$ 2613.7 [M−H]$^-$; found 2613.8; Products from T_5A(EU)AT_5; pAT_5 (P1), calcd $C_{60}H_{78}N_{15}O_{41}P_6$ 1851.2 [M−H]$^-$; found 1850.9; T_5ApR (P2), calcd $C_{66}H_{89}N_{16}O_{43}P_6$ 1980.3 [M−H]$^-$; found 1980.2; Products from T_5C(EU)CT_5; pCT_5 (P1), calcd $C_{59}H_{78}N_{13}O_{42}P_6$ 1827.2 [M−H]$^-$; found 1827.0; T_5CpR (P2), calcd $C_{65}H_{89}N_{14}O_{44}P_6$ 1956.3 [M−H]$^-$; found 1956.3; Products from T_5G(EU)GT_5; pGT_5 (P1), calcd $C_{60}H_{78}N_{15}O_{42}P_6$ 1867.2 [M−H]$^-$; found 1867.2; T_5GpR (P2), calcd $C_{66}H_{89}N_{16}O_{44}P_6$ 1996.3 [M−H]$^-$; found 1996.2; Products from CGCA_2T(EU)TA_2CGC; pTA_2CGC (P1), calcd $C_{58}H_{74}N_{23}O_{36}P_6$ 1855.2 [M−H]$^-$; found 1855.0; CGCA_2TpR (P2), calcd $C_{64}H_{85}N_{24}O_{38}P_6$ 1984.3 [M−H]$^-$; found 1984.3. R ($C_6H_{11}NO_2$) was calculated on the supposition that the cleavage reaction was accompanied by elimination of EU and addition of methylamine and OH^-.
