# Supplementary material for: Non-Enzymatic DNA Cleavage Reaction Induced by 5-Ethynyluracil in Methylamine Aqueous Solution and Application to DNA Concatenation
Source: PLoS One. 2014 Mar 19;9(3):e92369. doi: 10.1371/journal.pone.0092369 (PMC3960239; doi:10.1371/journal.pone.0092369)
Supplement: Method S1 — Preparation of DNA oligonucleotides containing 5-ethynyluracil (EU). (PDF) [file pone.0092369.s008.pdf]

Method S1

Preparation of DNA oligonucleotides containing 5-ethynyluracil (EU)

DNA oligonucleotides containing EU were synthesized on the DNA synthesizer by using the standard phosphoramidite method. The phosphoramidite to incorporate EU into DNA oligonucleotides was synthesized according to the literature [12,13]. Phosphoramidites of natural DNA nucleosides (^{Bz}dA, ^{Ac}dC, ^{dmf}dG, and T) were purchased from Glen research. The coupling time of EU phosphoramidite was extended to 30 minutes to ensure high coupling efficiency. The last DMTr group was not deprotected on the DNA synthesizer (DMTr-ON). After automated DNA synthesis, the CPG-support was put into a screw-cap tube and 1 mL of 50 mM K_2CO_3/MeOH was added into the tube. The suspension was left at 25°C for 16 hours or 65°C for 4 hours. A small amount of H_2O was added to the suspension and MeOH in the suspension was removed by speed-vac. H_2O was added to the residue to reach a total volume of ≈500 μL and 28% NH_3aq (500 μL) was added to the suspension. The suspension was left at 25°C for 16 hours. NH_3 in the suspension was removed by speed-vac. The suspension was passed through a 0.45 μm filter. The DMTr-ON DNA oligonucleotides were purified by reversed-phase HPLC with a linear gradient over 20 minutes from 5 to 50% CH_3CN in 50 mM ammonium formate (AF). After speed-vac and freeze-drying to remove the solvent and AF, the DMTr group on the DNA oligonucleotides was removed in 80% AcOH aqueous solution (100 μL) at 25°C for 30 minutes. H_2O was added to the solution to reach a total volume of ≈1 mL. The DNA oligonucleotides were purified by reversed-phase HPLC with a linear gradient over 20 minutes from 5 to 20% CH_3CN in 50 mM AF. The purified DNA oligonucleotides were desalted and identified by MALDI TOF mass spectrometry except for long primers (≥ 30 mer): T_6(EU)T_6, calcd for $C_{131}H_{167}N_{26}O_{89}P_{12}$ 3901.5 [M−H]$^-$; found 3901.8; (EU)$T_2AT_2GT_2$, calcd for $C_{91}H_{113}N_{24}O_{58}P_8$ 2718.8 [M−H]$^-$; found 2718.9; $T_2AT_2GT_2$(EU)T, calcd for $C_{101}H_{126}N_{26}O_{65}P_9$ 3023.0 [M−H]$^-$; found 3023.4; T_5A(EU)AT_5, calcd for $C_{131}H_{165}N_{32}O_{85}P_{12}$ 3919.6 [M−H]$^-$; found 3920.3; T_5C(EU)CT_5, calcd for $C_{129}H_{165}N_{28}O_{87}P_{12}$ 3871.5 [M−H]$^-$; found 3872.0; T_5G(EU)GT_5, calcd for $C_{131}H_{165}N_{32}O_{87}P_{12}$ 3951.6 [M−H]$^-$; found 3952.5; CGCA_2T(EU)TA_2CGC, calcd for $C_{127}H_{157}N_{48}O_{75}P_{12}$ 3927.6 [M−H]$^-$; found 3928.1; AG_2TGCT_3A(EU)GACTCT-GC_2G, calcd for $C_{206}H_{257}N_{74}O_{128}P_{20}$ 6437.1 [M−H]$^-$; found 6437.8.
