# Supplementary material for: Non-Enzymatic DNA Cleavage Reaction Induced by 5-Ethynyluracil in Methylamine Aqueous Solution and Application to DNA Concatenation
Source: PLoS One. 2014 Mar 19;9(3):e92369. doi: 10.1371/journal.pone.0092369 (PMC3960239; doi:10.1371/journal.pone.0092369)
Supplement: Method S2 — PCR amplification by using primers containing 5-ethynyluracil (EU). (PDF) [file pone.0092369.s009.pdf]

Methods S2

PCR amplification by using primers containing 5-ethynyluracil (EU)

KOD Fx neo (TOYOBO) was used for PCR. Before PCR, $CGCA_2T(EU)TA_2CGC$ in ×1 buffer for KOD Fx neo was heated with a temperature program, 94°C, 2 min → [98°C, 10 sec → 60°C, 30 sec → 68°C, 90 sec] ×30 → 4°C. After heating, the sample was directly analyzed by reversed-phase HPLC with a linear gradient over 20 minutes from 5 to 20% CH_3CN in 50 mM AF (Fig. S5). PCR solution (50 µL) contained primers (0.4 µM, each), dNTPs (400 µM, each), buffer (×1), KOD Fx neo (1 U), and template DNA, EcoRI-digested pBluescript-sk(–) (200 ng) or linear λ phage DNA (1 ng). The linear λ phage DNA (N3011) was purchased from New England Biolabs. DNA fragments for the construction of the 3.7 kbp plasmid were amplified using a temperature program, 94°C, 2 min → [98°C, 10 sec → 60°C, 30 sec → 68°C, 90 sec] ×30 → 4°C. The PCR-amplified DNA fragments were analyzed on a 1% agarose gel containing ethidium bromide in TAE buffer by electrophoresis.
