# Supplementary material for: Non-Enzymatic DNA Cleavage Reaction Induced by 5-Ethynyluracil in Methylamine Aqueous Solution and Application to DNA Concatenation
Source: PLoS One. 2014 Mar 19;9(3):e92369. doi: 10.1371/journal.pone.0092369 (PMC3960239; doi:10.1371/journal.pone.0092369)
Supplement: Method S3 — Preparation and cleavage of DNA oligonucleotides containing DNA base analogues. (PDF) [file pone.0092369.s010.pdf]

Methods S3

DNA base analogues for DNA cleavage

We found that the DNA base analogues described below can cause the same DNA cleavage as that induced by EU. Their synthetic schemes are shown following their respective synthetic procedures.

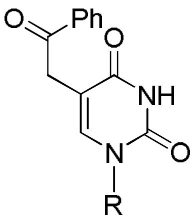

T1

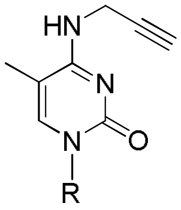

C1

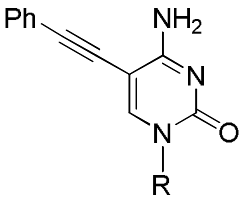

C2

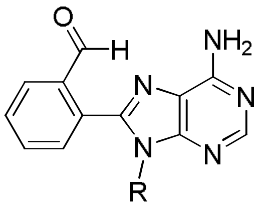

A1

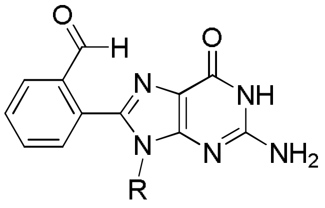

G1

Preparation of T_6(T1)T_6

T_6(T1)T_6 was postsynthetically prepared from the DNA oligonucleotide containing 5-ethynyluracil (PU) [17]. $AgNO_3$aq (100 mM, 200 μL) was added to T_6(PU)T_6 aqueous solution (1 mM strand concentration, 200 μL) [18]. The solution was mixed and left at 25°C for 16 hours. After addition of NaOHaq (10 M, 20 μL) [19], the solution was mixed and left at 25°C for 2 hours. H_2O (600 μL) containing acetic acid (20 μL) was added to the solution. The solution was filtered with 0.45 nm filter. The product was purified by reversed-phase HPLC with a linear gradient over 20 minutes from 8 to 20% CH_3CN/50 mM AF. The retention time of the product was 12.5 minutes. After desalination, the product was identified by MALDI TOF mass spectrometry: T_6(T1)T_6, calcd for $C_{137}H_{173}N_{26}O_{90}P_{12}$ 3995.6 $[M-H]^-$; found 3995.6.

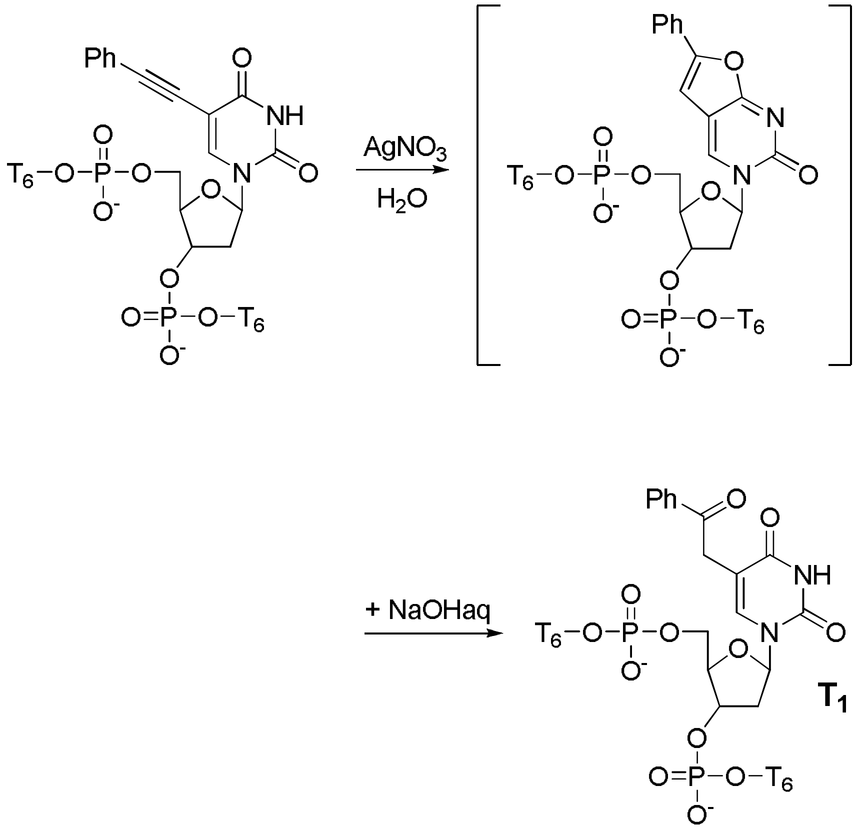

6-*N*-(3-propynyl)-5-methyl-2'-deoxycytidine (1)

Thymidine (1.21 g, 5.00 mmol) was suspended in CH_3CN (20 mL). Triethylamine (5.58 mL, 40.0 mmol) and acetic anhydride (1.89 mL, 20.0 mmol) were added to the suspension. After stirring at 25°C for 21 hours, methanol (10 mL) was added to the solution and stirred for 2 hours. The solvent was removed by evaporation. AcOEt (100 mmL) and saturated NaClaq (100 mL) were added to the residue and the product was extracted to the organic layer. The organic layer was washed with saturated NaClaq (100 mL) twice, dried over magnesium sulfate, and filtered. The solvent was removed by evaporation and the residue was dried under reduced pressure. 1,2,4-triazole (3.45 g, 50 mmol), CH_3CN (30 mL), and triethylamine (7.67 mL, 55.0 mmol) were added to the residue. $POCl_3$ (932 μL, 10.0 mmol) was slowly added to the solution under constant stirring. After stirring for 1 hour, the solvent was removed by evaporation. AcOEt (100 mmL) and saturated NaClaq (100 mL) were added to the residue and the product was extracted to the organic layer. The organic layer was washed with saturated NaClaq (100 mL) twice, dried over magnesium sulfate, and filtered. The solvent was removed by evaporation and the residue was dried under reduced pressure

[20]. 1,4-dioxane (30 mL) was added to the residue and the solution was stirred. Propargylamine (961 µL, 15.0 mmol) was added to the solution. After stirring at 25°C for 2 hours, methanol (30 mL) and 28% NH_3aq (30 mL) were added to the solution. The solution was stirred at 25°C for 24 hours. The solvent was removed by evaporation and the product **1** was purified by silica gel column chromatography (10–15% MeOH/CH_2Cl_2). The product **1** was obtained as a white powder (873 mg, 3.13 mmol, 63%): ^{1}H NMR (DMSO-d6) *δ* 7.65 (d, *J* = 0.9, 1H), 7.57 (t, *J* = 5.7, 1H), 5.19 (d, *J* = 4.6, 1H), 5.01 (t, *J* = 5.3, 1H), 4.23-4.18 (m, 1H), 4.14-4.01 (m, 2H),3.76-3.74 (m, 1H),3.62-3.50 (m, 2H), 3.04 (t, *J* = 2.5 , 1H),2.12-2.05 (m, 1H),1.99-1.92 (m, 1H),1.84 (s, 3H); ^{13}C NMR (DMSO-d6) *δ* 162.4, 154.9, 138.0, 101.7, 87.2, 84.8, 81.5, 72.6, 70.4, 61.4, 40.3, 29.4, 13.0; HRMS (ESI) calcd for $C_{13}H_{17}N_3O_4Na$ ($[M+Na]^+$) 302.1116, found 302.1117.

5'-*O*-dimetoxytrityl-6-*N*-(3-propynyl)-5-methyl-2'-deoxycytidine (2)
Pyridine (10 mL) was added to **1** (312 mg, 1.12 mmol) and DMTrCl (454 mg, 1.34 mmol). The solution was stirred at 25°C for 1 hour. After addition of methanol (10 mL), the solvent was removed by evaporation. The residual pyridine was removed by coevaporation with CH_2Cl_2 and hexane. The product **2** was purified by silica gel column chromatography (2–5% MeOH, 2% Et_3N/CH_2Cl_2). The product **2** was obtained as a white powder (631 mg, 0.924 mmol, 83%): ^{1}H NMR (DMSO-d6) *δ* 7.63 (t, *J* = 5.7, 1H), 7.51 (s, 1H), 7.39-7.35 (m, 2H),7.32-7.19 (m, 7H), 6.90-6.86 (m, 4H),6.21 (t, *J* = 6.6, 1H), 5.34 (d, *J* = 4.1, 1H), 4.31-4.25 (m, 1H), 4.14-4.02 (m, 2H),3.90-3.86 (m, 1H),3.72 (s, 6H), 3.22-3.16 (m, 2H),3.09-3.00 (m, 6H),2.21-2.06 (m, 2H),1.50 (s, 3H), 1.23-1.15 (m, 9H); ^{13}C NMR (DMSO-d6) *δ* 162.3, 158.1, 157.9, 154.8, 144.7, 137.4, 135.5, 135.3, 130.0, 127.9, 127.7, 126.8, 113.2, 113.0, 101.9, 85.8, 85.4, 84.6, 81.4, 72.6, 70.5, 63.6, 55.0, 45.3, 29.4, 8.4; HRMS (ESI): calcd for $C_{34}H_{35}N_3O_6Na$ ($[M+Na]^+$) 604.2525, found 604.2424.

Preparation of T_6(C1)T_6
2 (291 mg, 0.426 mmol) was dissolved in CH_3CN (5 mL). Disopropylethylamine (261 µL, 1.50 mmol) and 2-cyanoethyl *N,N*-diisopropylchlorophosphoramidite (223 µL, 1.00 mmol) was added to the solution. The solution was stirred at 25°C for 30 minutes. AcOEt (25 mL) and saturated NaClaq (25 mL) were added to the suspension. The product was extracted to the organic layer. The organic layer was washed with saturated NaClaq (25 mL) twice, dried over magnesium sulfate,

and filtered. The solvent was removed by evaporation and the residue was dried by coevaporation with acetonitrile and further dried under reduced pressure. After dissolving the residue to CH_3CN (10 mL), the phosphoramidite solution was used directly for automated DNA synthesis. T_6(C1)T_6 was synthesized by standard phosphoramidite method on the DNA synthesizer. The protected oligonucleotide was cleaved by 28% NH_3aq from the solid support and deprotected at 25°C for 16 hours in 28% NH_3aq. After removal of ammonia by speed-vac, the aqueous solution was filtered through 0.45 μm filter. T_6(C1)T_6 was purified by reversed-phase HPLC with a linear gradient over 20 minutes from 0 to 50% CH_3CN/50 mM AF. The retention time of the product was 10.6 minutes. After the desalination, the product was identified by MALDI TOF mass spectrometry: T_6(C1)T_6, calcd for $C_{133}H_{172}N_{27}O_{88}P_{12}$ 3928.6 $[M-H]^-$; found 3928.8.

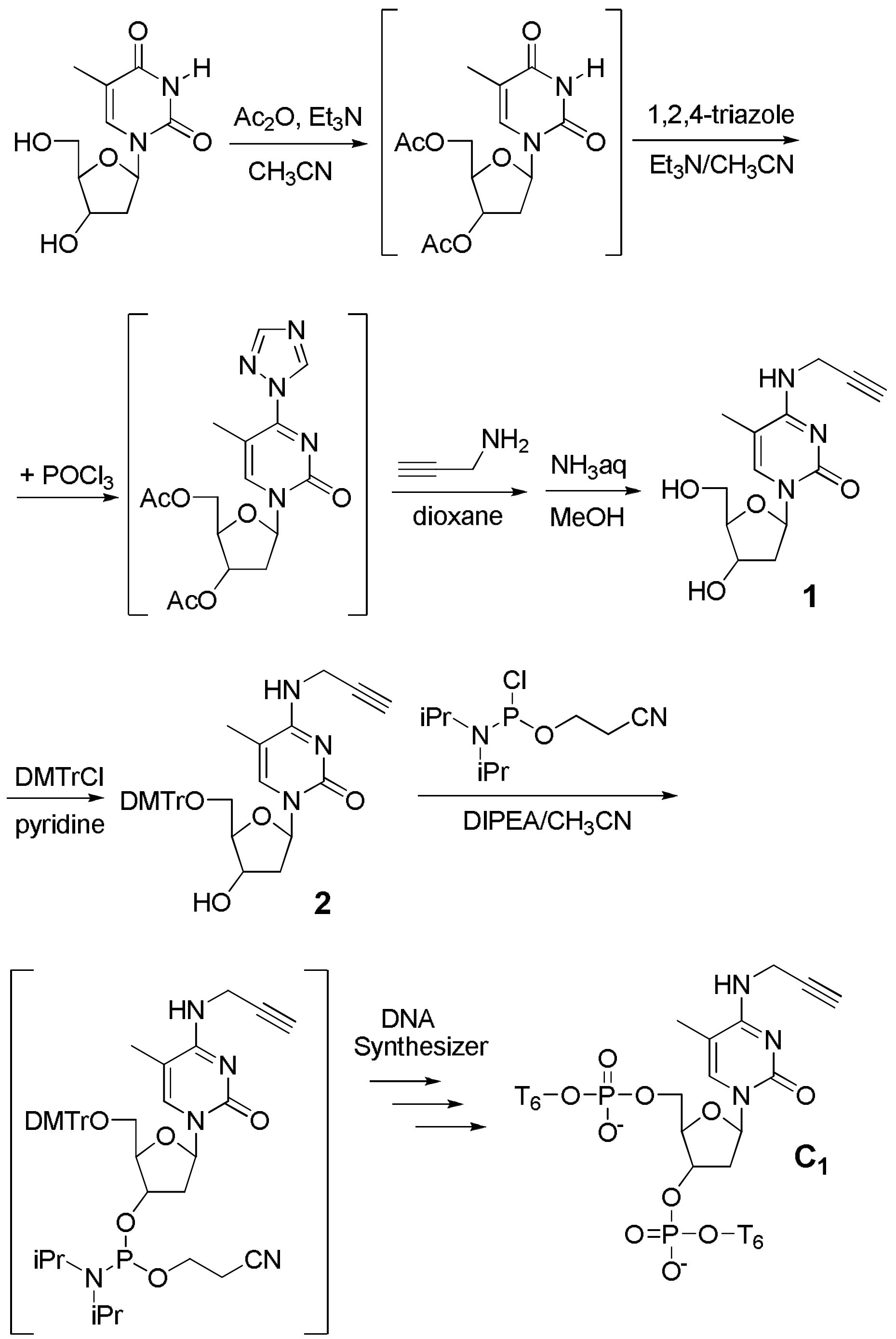

4-*N*-[*N*,*N*-di(*n*-butyl)aminomethylene]-5-phenylethynyl-2'-deoxycytidine (3)
5-phenylethynyl-2'-deoxycytidine (708 mg, 2.16 mmol) [21] was dissolved in methanol (10 mL). *N*,*N*-di(*n*-butyl)formamide dimethyl acetal (2.0 mL, 8.6 mmol) [22] was added to the solution. The solution was stirred at 25°C for 2 hours. The solvent was removed by evaporation. The product **3** was purified by silica gel column chromatography (0–10% MeOH/CH_2Cl_2). The product **3** was obtained as

a pale brown oil (700 mg, 1.50 mmol, 69%): ^{1}H NMR (DMSO-d6) δ 8.64 (s, 1H), 8.44 (s, 1H), 7.43-7.34 (m, 5H), 6.12 (t, J = 6.2, 1H), 5.23 (d, J = 4.1, 1H), 5.13 (t, J = 4.8, 1H), 4.26-4.21 (m, 1H), 3.84-3.81 (m, 1H), 3.70-3.44 (m, 6H), 2.26-2.20 (m, 1H), 2.08-2.01 (m, 1H), 1.66-1.52 (m, 4H), 1.32-1.22 (m, 4H), 0.90 (t, J = 7.3, 3H), 0.82 (t, J = 7.3, 3H); ^{13}C NMR (DMSO-d6) δ 169.7, 157.4, 153.4, 145.3, 130.7, 128.6, 128.1, 123.2, 97.5, 91.6, 87.6, 85.8, 84.5, 69.7, 60.7, 54.9, 51.4, 45.4, 41.0, 30.4, 28.5, 19.6, 19.1, 13.5; HRMS (ESI) calcd for $C_{26}H_{35}N_4O_4$ ($[M+H]^+$) 467.2658, found 467.2653.

5'-*O*-dimethoxytrityl-4-*N*-[*N,N*-di(*n*-butyl)aminomethylene]-5-phenylethynyl -2'-deoxycytidine (4)

Pyridine (10 mL) was added to **3** (700 mg, 1.50 mmol) and DMTrCl (763 mg, 2.25 mmol). The solution was stirred at 25°C for 1 hour. After addition of methanol (10 mL), the solvent was removed by evaporation. The residual pyridine was removed by coevaporation with CH_2Cl_2 and hexane. The product **4** was purified by silica gel column chromatography (0–5% MeOH, 2% Et_3N/CH_2Cl_2). The product **4** was obtained as white powder (924 mg, 1.20 mmol, 80%): ^{1}H NMR (DMSO-d6) δ 8.65 (s, 1H), 8.17 (s, 1H), 7.44-6.81 (m, 18H), 6.14 (t, J = 6.6, 1H), 5.32 (d, J = 4.6, 1H), 4.31-4.26 (m, 1H), 4.02-3.97 (m, 1H), 3.644 (s, 3H), 3.637 (s, 3H), 3.57-3.49 (m, 4H), 3.23-3.16 (m, 2H), 2.36-2.30 (m, 1H), 2.17-2.10 (m, 1H), 1.66-1.53 (m, 4H), 1.32-1.22 (m, 4H), 0.91 (t, J = 7.3, 3H), 0.82 (t, J = 7.3, 3H); ^{13}C NMR(DMSO-d6) δ 169.7, 158.03, 158.01, 157.5, 153.4, 144.7, 144.3, 135.6, 135.4, 130.6, 129.7, 129.6, 128.3, 127.93, 127.86, 127.6, 126.6, 123.0, 113.2, 97.9, 91.7, 86.2, 85.9, 83.9, 70.6, 63.5, 54.9, 54.5, 45.4, 41.2, 30.4, 28.5, 19.6, 19.1, 13.5; HRMS (ESI): calcd for $C_{47}H_{53}N_4O_6$ ($[M+H]^+$) 769.3965, found 769.3985.

Preparation of T_6(C2)T_6

4 (109 mg, 0.142 mmol) was dissolved in CH_3CN (5 mL). Triethylamine (157 μL, 1.13 mmol) and 2-cyanoethyl *N,N*-diisopropylchlorophosphoramidite (126 μL, 0.565 mmol) was added to the solution. The solution was stirred at 25°C for 30 minutes. AcOEt (25 mL) and saturated NaClaq (25 mL) were added to the suspension. The product was extracted to the organic layer. The organic layer was washed with saturated NaClaq (25 mL) twice, dried over magnesium sulfate, and filtered. The solvent was removed by evaporation and the residue was dried by coevaporation with acetonitrile and further dried under reduced pressure.

After dissolving the residue to CH_3CN (4.7 mL), the phosphoramidite solution was used directly for automated DNA synthesis. T_6(C2)T_6 was synthesized by the standard phosphoramidite method on the DNA synthesizer. The last DMTr group was not deprotected on the DNA synthesizer. The protected oligonucleotide was cleaved by 28% NH_3aq from the solid support and deprotected at 25°C for 16 hours in 28% NH_3aq. After removal of ammonia by speed-vac, the aqueous solution was filtered through a 0.45 μm filter. 5'-DMTr-T_6(C2)T_6 was purified by reversed-phase HPLC with a linear gradient over 20 minutes from 5 to 50% CH_3CN/50 mM AF. The retention time of the 5'-DMTr-T_6(C2)T_6 was 15.2 minutes. After speed-vac and freeze-drying to remove the solvent and AF, the DMTr group was removed at 25°C for 30 minutes in 80% AcOHaq (100 μL). The solution was diluted with H_2O to be ≈1 mL and passed through a 0.45 m filter. T_6(C2)T_6 was purified by reversed-phase HPLC with a linear gradient over 20 minutes from 8 to 20% CH_3CN/50 mM AF. The retention time of the product was 13.6 minutes. After the desalination, the product was identified by MALDI TOF mass spectrometry: T_6(C2)T_6, calcd for $C_{137}H_{172}N_{27}O_{88}P_{12}$ 3976.6 $[M-H]^-$; found 3977.1.

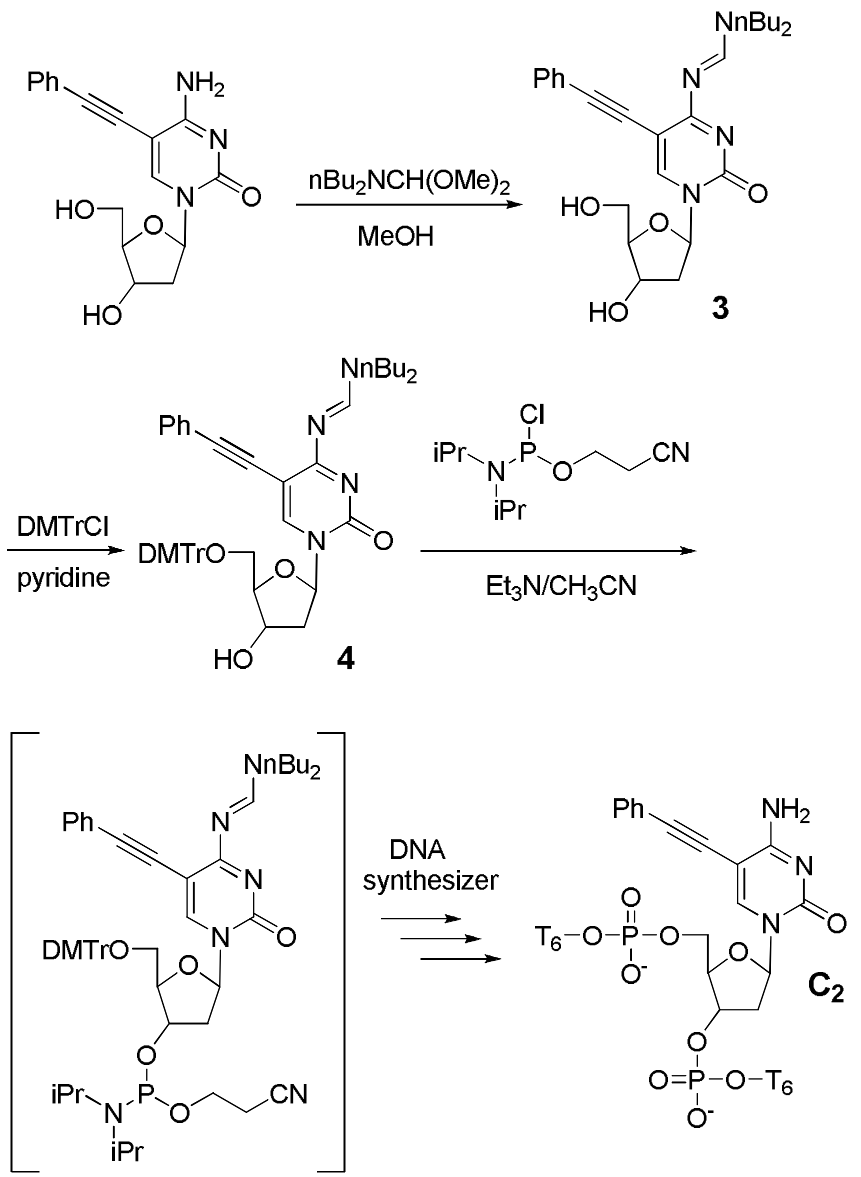

8-(2-formylphenyl)-2'-deoxyadenosine (5)

8-bromo-2'-deoxyadenosine (2.20 g, 6.66 mmol) [23], 2-formylbenzene boronic acid (2.00 g, 13.3 mmol), palladium (II) acetate (150 mg, 0.666 mmol), triphenylphosphine (525 mg, 2.00 mmol), and potassium carbonate (1.84 g, 13.3 mmol) were stirred in acetonitrile (40 mL) and H_2O (20 mL) at 80°C for 4 hours [24,25]. The solvent was removed by evaporation. Methanol (200 mL) was added to the residue. The resulting solution was filtered. The solvent was removed by evaporation. The product **5** was purified by silica gel column chromatography (10% MeOH/CH_2Cl_2). The product **5** was obtained as white powder (1.81 g, 5.09 mmol, 76%): ^{1}H NMR (DMSO-d6) δ 9.93 (s, 1H), 8.18 (s,

1H), 8.09-8.07 (m, 1H), 7.90-7.80 (m, 2H), 7.74-7.72 (m, 1H), 7.46 (br, 2H), 5.90 (dd, *J* = 8.5, 6.2, 1H), 5.49 (dd, *J* = 8.3, 4.1, 1H), 5.17 (d, *J* = 4.1, 1H), 4.39-4.35 (m, 1H), 3.82-3.79 (m, 1H), 3.65-3.59 (m, 1H), 3.50-3.44 (m, 1H), 3.19 (ddd, *J* = 12.8, 8.7, 6.0, 1H), 2.06 (ddd, *J* = 13.0, 6.2, 1.8, 1H); ^{13}C NMR (DMSO-d6) δ 191.7, 156.2, 152.3, 149.6, 147.4, 135.4, 133.8, 131.5, 131.4, 130.8, 129.5, 119.3, 88.3, 85.7, 71.3, 62.2, 37.4; HRMS (ESI) calcd for $C_{17}H_{18}N_5O_4$ ($[M+H]^+$) 356.1359, found 356.1364.

6-*N*-[*N*,*N*-di(*n*-butyl)aminomethylene]-8-(2-formylphenyl)-2'-deoxyadenosine (6)

5 (1.55 g, 4.36 mmol) was dissolved in methanol (20 mL). *N*,*N*-di(*n*-butyl)-formamide dimethyl acetal (8.14 mL, 34.8 mmol) was added to the solution [22]. The solution was stirred at 25°C for 2 hours. The solvent was removed by evaporation. The product **6** was purified by silica gel column chromatography (2–5% MeOH/CH_2Cl_2). The product **6** was obtained as a pale brown oil (1.40 g, 2.83 mmol, 65%): ^{1}H NMR (DMSO-d6) δ 9.92 (s, 1H), 8.91 (s, 1H), 8.45 (s, 1H), 8.09 (d, *J* = 7.8, 1H), 7.91-7.82 (m, 2H), 7.74 (d, *J* = 7.3, 1H), 5.94 (dd, *J* = 7.8, 6.8, 1H), 5.28 (dd, *J* = 7.6, 4.4, 1H), 5.18 (d, *J* = 4.1, 1H), 4.40-4.36 (m, 1H), 3.81-3.77 (m, 1H), 3.64-3.41 (m, 6H), 3.25-3.18(m, 1H), 2.09-2.04 (m, 1H), 1.62-1.52 (m, 4H), 1.33-1.22 (m, 4H), 0.94-0.83 (m, 6H); ^{13}C NMR(DMSO-d6) δ 191.8, 159.5, 157.9, 151.7, 151.6, 149.3, 135.5, 133.8, 131.5, 131.3, 130.9, 129.7, 125.8, 88.2, 85.5, 71.2, 62.1, 50.9, 44.4, 37.2, 30.4, 28.7, 19.6, 19.1,13.7, 13.5; HRMS (ESI) calcd for $C_{26}H_{35}N_6O_4$ ($[M+H]^+$) 495.2720, found 495.2718.

5'-*O*-dimethoxytrityl-6-*N*-[*N*,*N*-di(*n*-butyl)aminomethylene]-8-(2-formylphenyl)-2'-deoxyadenosine (7)

Pyridine (10 mL) was added to **6** (1.31 g, 2.64 mmol) and DMTrCl (991 mg, 2.92 mmol). The solution was stirred at 25°C for 1 hour. After addition of methanol (10 mL), the solvent was removed by evaporation. The residual pyridine was removed by coevaporation with CH_2Cl_2 and hexane. The product **7** was purified by silica gel column chromatography (2% MeOH, 2% Et_3N/CH_2Cl_2). The product **7** was obtained as a white powder (1.24 g, 1.56 mmol, 59%): ^{1}H NMR (DMSO-d6) δ 9.89 (s, 1H), 8.88 (s, 1H), 8.25 (s, 1H), 8.06-8.04 (m, 1H), 7.88-7.78 (m, 3H), 7.33-7.31 (m, 2H), 7.21-7.15 (m, 7H), 6.81-6.73 (m, 4H), 5.98 (t, *J* = 6.9, 1H), 5.25 (d, *J* = 4.6, 1H), 4.57-4.53 (m, 1H), 3.94-3.90 (m, 1H), 3.71 (s, 3H), 3.69 (s, 3H), 3.59-3.53 (m, 2H), 3.44-3.32 (m, 3H), 3.20-3.12 (m, 2H),

2.11 (ddd, J = 13.0, 7.3, 4.3, 1H), 1.62-1.52 (m, 4H), 1.34-1.23 (m, 4H), 0.90-0.87 (m, 6H); ^{13}C NMR(DMSO-d6) δ 191.6, 159.2, 157.9, 157.9, 157.7, 151.8, 149.5, 145.0, 135.7, 135.60, 135.55, 133.6, 131.7, 131.4, 130.7, 129.7, 129.5, 129.1, 127.6, 126.5, 125.6, 113.0, 112.9, 85.8, 85.2, 84.8, 71.0, 63.6, 54.94, 54.90, 50.9, 44.4, 36.3, 30.4, 28.7, 19.6, 19.1, 13.7, 13.5; HRMS (ESI): calcd for $C_{47}H_{53}N_6O_6$ ($[M+H]^+$) 797.4027, found 797.4046.

Preparation of T_6(A1)T_6

7 (159 mg, 0.200 mmol) was dissolved in CH_3CN (5 mL). Triethylamine (223 μL, 1.60 mmol) and 2-cyanoethyl *N,N*-diisopropylchlorophosphoramidite (178 μL, 0.800 mmol) were added to the solution. The solution was stirred at 25°C for 30 minutes. AcOEt (25 mL) and saturated NaClaq (25 mL) were added to the suspension. The product was extracted to the organic layer. The organic layer was washed with saturated NaClaq (25 mL) twice, dried over magnesium sulfate, and filtered. The solvent was removed by evaporation and the residue was dried by coevaporation with acetonitrile and further dried under reduced pressure. After dissolving the residue in CH_3CN (10 mL), the phosphoramidite solution was used directly for automated DNA synthesis. T_6(A1)T_6 was synthesized by the standard phosphoramidite method on the DNA synthesizer. The last DMTr group was not deprotected on the DNA synthesizer. The protected oligonucleotide was cleaved from the solid support using 28% NH_3aq and deprotected at 25°C for 16 hours in 28% NH_3aq. After removal of ammonia by speed-vac, the aqueous solution was filtered through a 0.45 μm filter. 5'-DMTr-T_6(C2)T_6 was purified by reversed-phase HPLC with a linear gradient over 20 minutes from 0 to 50% CH_3CN/50 mM AF. The retention time of the 5'-DMTr-T_6(A1)T_6 was 16.5 minutes. After speed-vac and freeze-drying to remove the solvent and AF, the DMTr group was removed at 25°C for 30 minutes in 80% AcOHaq (100 μL). The solution was diluted with H_2O to 1 mL and filtered through a 0.45 m filter. T_6(C2)T_6 was purified by reversed-phase HPLC with a linear gradient over 20 minutes from 5 to 20% CH_3CN/50 mM AF. The retention time of the product was 14.9 minutes. After the desalination, the product was identified by MALDI TOF mass spectrometry: T_6(A1)T_6, calcd for $C_{137}H_{172}N_{29}O_{88}P_{12}$ 4004.7 $[M-H]^-$; found 4005.2.

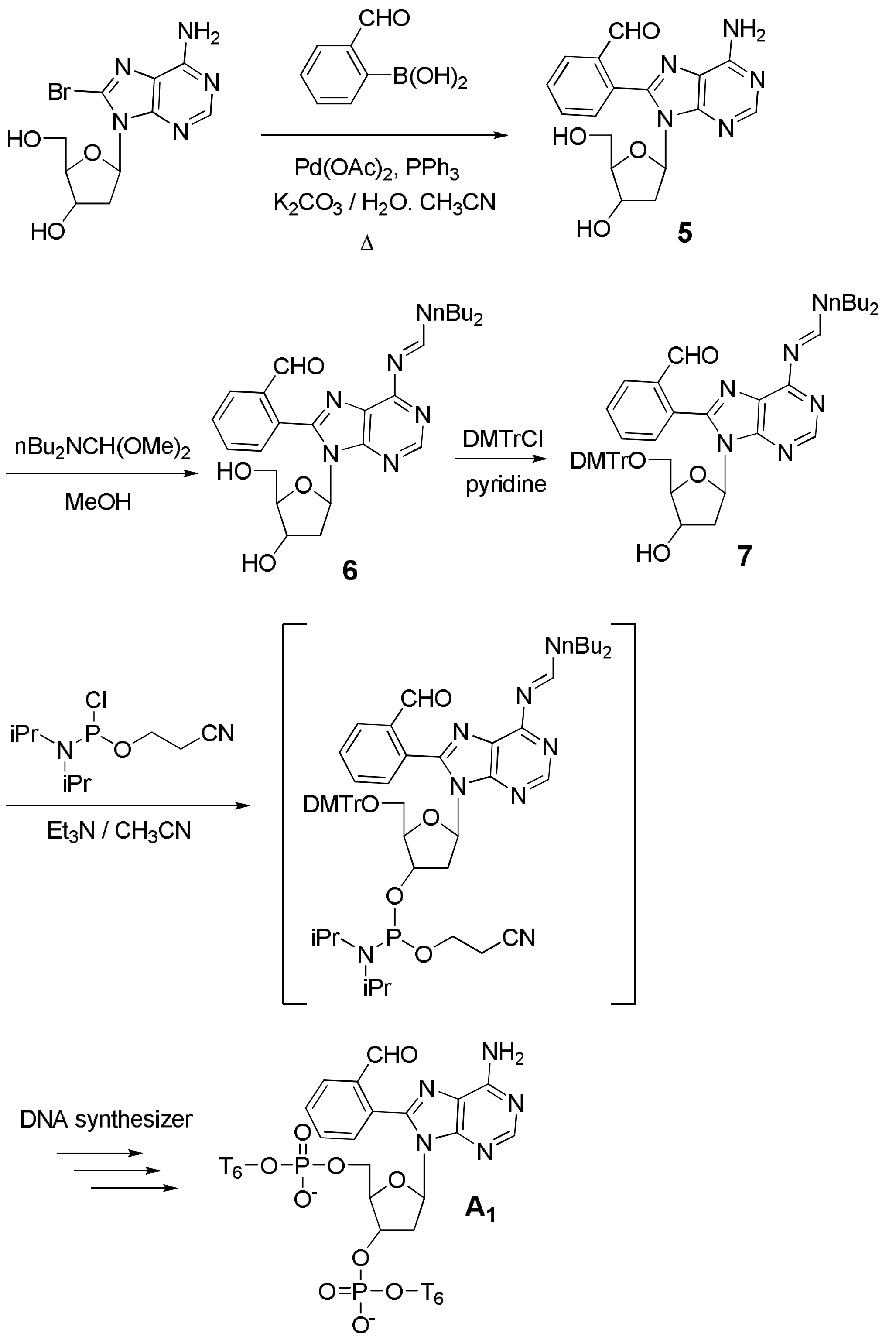

8-(2-formylphenyl)-2'-deoxyguanosine (8)

8-bromo-2'-deoxyguanosine (3.46 g, 10.0 mmol) [26], 2-formylbenzene boronic acid (3.00 g, 20.0 mmol), palladium (II) acetate (224 mg, 1.00 mmol),

triphenylphosphine (787 mg, 3.00 mmol), and potassium carbonate (2.76 g, 20 mmol) were stirred in acetonitrile (80 mL) and H_2O (40 mL) at 80°C for 4 hours [24,25]. The solvent was removed by evaporation. Methanol (200 mL) was added to the residue. The resultant solution was filtered. The solvent was removed by evaporation. The product **8** was purified by silica gel column chromatography (10–20% MeOH/CH_2Cl_2). The product **8** was obtained as a pale yellow powder (2.97 g, 8.00 mmol, 80%): ^{1}H NMR (DMSO-d6) δ 10.80 (br, 1H), 9.85 (s, 1H), 7.98 (dd, J = 7.8, 1.4, 1H),7.84-7.80 (m, 1H), 7.75-7.67 (m, 2H), 6.47 (br, 2H), 5.92 (dd, J = 8.2, 6.4, 1H), 5.10 (d, J = 4.1 Hz, 1H), 4.89-4.86 (m, 1H), 4.21-4.18 (m, 1H), 3.72-3.69 (m, 1H), 3.49-3.38 (m, 2H), 2.98 (ddd, J = 13.2, 8.2, 5.9, 1H), 4.20 (ddd, J = 13.3, 6.4, 2.3, 1H); ^{13}C NMR(DMSO-d6) δ 191.7, 156.5, 153.3, 151.8, 143.4, 135.5, 133.5, 132.5, 131.4, 130.1, 128.4, 117.5, 87.8, 84.6, 71.0, 61.9, 36.8; HRMS (ESI) calcd for $C_{17}H_{17}N_5O_4Na$ ($[M+Na]^+$) 394.1127, found 394.1115.

2-*N*-[*N*,*N*-di(*n*-butyl)aminomethylene]-8-(2-formylphenyl)-2'-deoxyguanosine (9)

8 (2.32 g, 6.25 mmol) was dissolved in methanol (20 mL). *N*,*N*-di(*n*-butyl)-formamide dimethyl acetal (11.7 mL, 50.0 mmol) was added to the solution [22]. The solution was stirred 25°C for 2 hours. The solvent was removed by evaporation. The product **9** was purified by silica gel column chromatography (5–10% MeOH/CH_2Cl_2). The product **9** was obtained as a pale yellow powder (2.10 g, 4.11 mmol, 66%): ^{1}H NMR (DMSO-d6) δ 11.51 (s, 1H), 9.89 (s, 1H), 8.53 (s, 1H), 8.02-8.00 (m, 1H), 7.87-7.83 (m, 1H), 7.77-7.73 (m, 1H), 7.69-7.67 (m, 1H), 5.92 (dd, J = 7.8, 6.9, 1H), 5.16 (d, J = 4.2, 1H), 4.85-4.82 (m, 1H), 4.33-4.29 (m, 1H), 3.76-3.73 (m, 1H), 3.58-3.35 (m, 6H), 3.14 (ddd, J = 13.7, 7.3, 6.4, 1H), 2.05 (ddd, J = 13.3, 6.4, 2.8, 1H), 1.63-1.54 (m, 4H), 1.35-1.23 (m, 4H), 0.94-0.89 (m, 6H); ^{13}C NMR(DMSO-d6) δ 191.7, 157.8, 157.4, 157.3, 150.5, 144.6, 135.5, 133.6, 132.1, 131.3, 130.2, 128.7, 120.5, 87.8, 85.0, 71.0, 62.0, 51.2, 44.9, 37.3, 30.3, 28.5, 19.6, 19.1, 13.7, 13.5; HRMS (ESI) calcd for $C_{26}H_{34}N_6O_5Na$ ($[M+Na]^+$) 533.2488, found 533.2481.

5'-*O*-dimethoxytrityl-2-*N*-[*N*,*N*-di(*n*-butyl)aminomethylene]-8-(2-formylphenyl)-2'-deoxyguanosine (10)

Pyridine (16 mL) was added to **9** (2.10 g, 4.11 mmol) and DMTrCl (1.53 g, 4.52 mmol). The solution was stirred at 25°C for 1 hour. After addition of methanol (10 mL), the solvent was removed by evaporation. The residual pyridine was

removed by coevaporation with CH_2Cl_2 and hexane. The product **10** was purified by silica gel column chromatography (80–98% AcOEt, 2% Et_3N/*n*-hexane). The product **10** was obtained as a pale yellow powder (2.20 g, 2.71 mmol, 66%): ^{1}H NMR (DMSO-d6) δ 11.52 (s, 1H), 9.88 (s, 1H), 8.38 (s, 1H), 7.98 (d, J = 7.8 Hz, 1H), 7.80-7.79 (m, 2H), 7.74-7.70 (m, 1H), 7.31-7.29 (m, 2H), 7.20-7.15 (m, 7H), 6.79-6.72 (m, 4H), 6.00 (dd, J = 8.0, 4.8 Hz, 1H), 5.19 (d, J = 5.1 Hz, 1H), 4.54-4.48 (m, 1H), 3.84-3.80 (m, 1H), 3.70 (s, 3H), 3.69 (s, 3H), 3.47-3.43 (m, 2H), 3.31-3.26 (m, 1H), 3.21-3.13 (m, 3H), 3.07-3.04 (m, 1H), 2.17 (ddd, J = 13.7, 8.0, 5.8 Hz, 1H), 1.59-1.51 (m, 2H), 1.45-1.36 (m, 2H), 1.33-1.24 (m, 2H), 1.15-1.09 (m, 2H), 0.93-0.89 (m, 3H), 0.79-0.75 (m, 3H); ^{13}C NMR(DMSO-d6) δ 191.6, 158.0, 157.9, 157.5, 157.3, 156.9, 150.3, 144.9, 144.8, 135.7, 135.6, 133.4, 132.5, 131.2, 130.1, 129.6, 129.4, 128.3, 127.6, 126.5, 120.4, 112.9, 85.4, 85.2, 84.0, 70.6, 63.6, 55.0, 54.9, 51.2, 45.0, 37.6, 30.2, 28.5, 19.6, 19.0, 13.7, 13.5; HRMS (ESI): calcd for $C_{47}H_{53}N_6O_7$ ($[M+H]^+$) 813.3976, found 813.3976.

Preparation of T_6(G1)T_6

10 (163 mg, 0.200 mmol) was dissolved in CH_3CN (5 mL). Triethylamine (223 µL, 1.60 mmol) and 2-cyanoethyl *N,N*-diisopropylchlorophosphoramidite (178 µL, 0.800 mmol) were added to the solution. The solution was stirred at 25°C for 30 minutes. AcOEt (25 mL) and saturated NaClaq (25 mL) were added to the suspension. The product was extracted to the organic layer. The organic layer was washed with saturated NaClaq (25 mL) twice, dried over magnesium sulfate, and filtered. The solvent was removed by evaporation and the residue was dried by coevaporation with acetonitrile and further dried under reduced pressure. After dissolving the residue in CH_3CN (10 mL), the phosphoramidite solution was used directly for automated DNA synthesis. T_6(G1)T_6 was synthesized by standard phosphoramidite method on the DNA synthesizer. The last DMTr group was not deprotected on the DNA synthesizer. The protected oligonucleotide was cleaved by 28% NH_3aq from the solid support and deprotected at 25°C for 16 hours in 28% NH_3aq. After removal of ammonia by speed-vac, the aqueous solution was filtered through a 0.45 µm filter. 5'-DMTr-T_6(G1)T_6 was purified by reversed-phase HPLC with a linear gradient over 20 minutes from 5 to 50% CH_3CN/50 mM AF. The retention time of the 5'-DMTr-T_6(G1)T_6 was 15.2 minutes. After speed-vac and freeze-drying to remove the solvent and AF, the DMTr group was removed at 25°C for 30 minutes in 80% AcOHaq (100 µL). The solution was diluted with H_2O to 1 mL and filtered through 0.45 m filter. T_6(C2)T_6

was purified by reversed-phase HPLC with a linear gradient over 20 minutes from 5 to 20% CH_3CN/50 mM AF. The retention time of the product was 14.6 minutes. After desalination, the product was identified by MALDI TOF mass spectrometry: T_6(G1)T_6, calcd for $C_{137}H_{172}N_{29}O_{89}P_{12}$ 4020.7 [M−H]$^-$; found 4022.0.

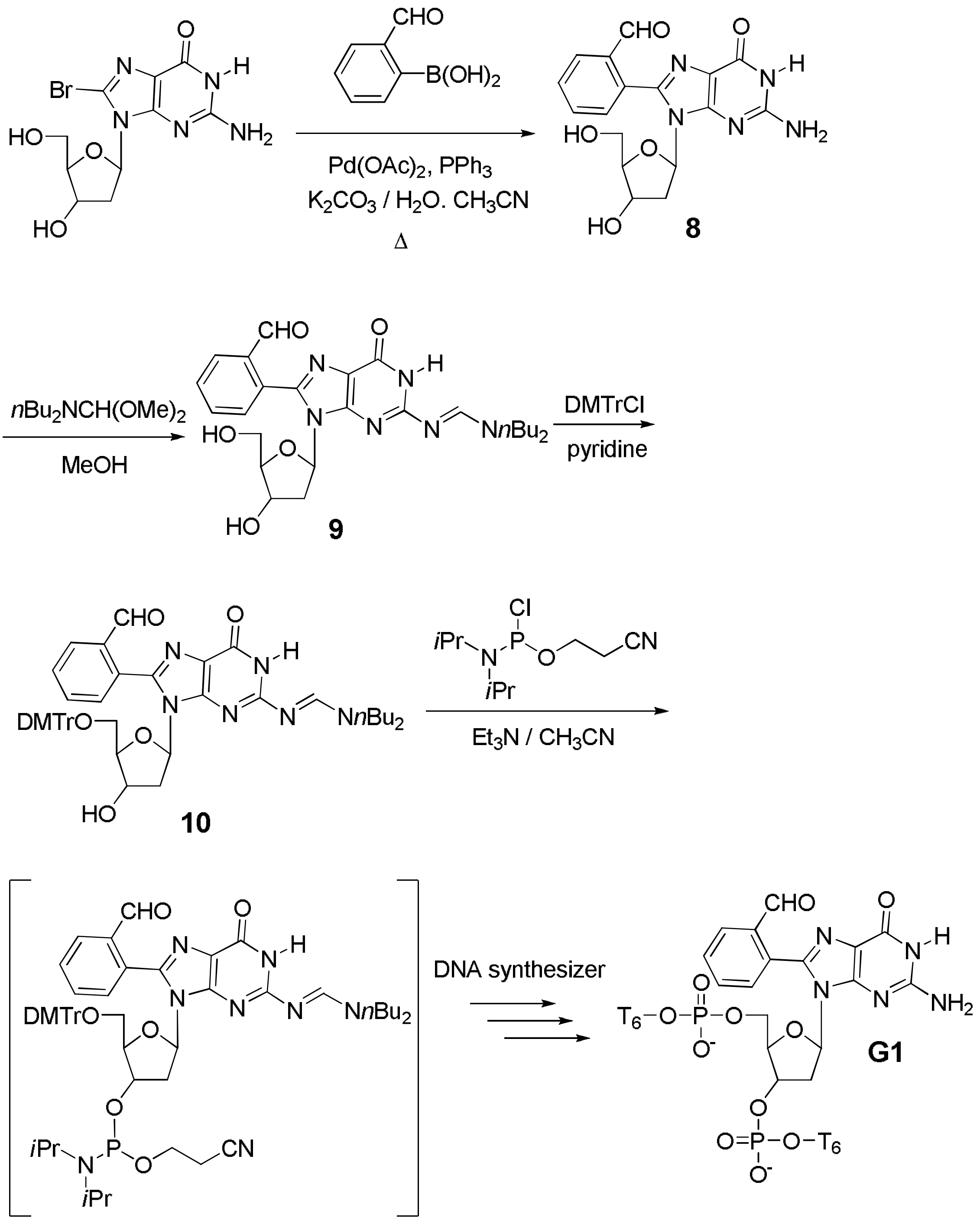

DNA cleavage reaction using designed DNA base analogues

DNA cleavage was carried out using an aqueous solution of DNA oligonucleotides (100 μM) in a screw-cap tube. The same volume of 40% methylamine was added to the sample and the resulting solution was heated at 70°C in a water bath. After removal of methylamine by speed-vac, the sample was directly analyzed by reversed-phase HPLC with a linear gradient over 20 min from 8% to 20% CH_3CN in 50 mM AF. The cleavage products, P1 and P2 were purified, desalted by the HPLC and identified by MALDI TOF mass spectrometry: P1 (pT_6), calcd for $C_{60}H_{79}N_{12}O_{43}P_6$ 1843.2 $[M-H]^-$; found 1841.6 ($P1(T_6(T1)T_6)$), 1841.8 ($P1(T_6(C1)T_6)$), 1842.0 ($P1(T_6(C2)T_6)$), 1842.0 ($P1(T_6(A1)T_6)$), and 1842.2 ($P1(T_6(G1)T_6)$); P2 (T_6pR), calcd for $C_{66}H_{90}N_{13}O_{45}P_6$ 1971.3 $[M-H]^-$; found 1971.0 ($P2(T_6(T1)T_6)$), 1971.4 ($P2(T_6(C1)T_6)$), 1971.1 ($P2(T_6(C2)T_6)$), 1971.2 ($P2(T_6(A1)T_6)$), and 1971.0 ($P2(T_6(G1)T_6)$).
