# Supplementary material for: Non-Enzymatic DNA Cleavage Reaction Induced by 5-Ethynyluracil in Methylamine Aqueous Solution and Application to DNA Concatenation
Source: PLoS One. 2014 Mar 19;9(3):e92369. doi: 10.1371/journal.pone.0092369 (PMC3960239; doi:10.1371/journal.pone.0092369)
Supplement: Method S4 — 5′-phosphorylation of the DNA oligonucleotide using 5-phenylethynyluracil (PU). (PDF) [file pone.0092369.s011.pdf]

Methods S4

5'-phosphorylation of the DNA oligonucleotide using 5-phenylethynyluracil (PU)

Here, we used 5-phenylethynyluracil (PU) instead of 5-ethynyluracil because of the more facile synthesis of the corresponding phosphoramidite [17]. DMTr-(PU)$T_2AT_2GT_2$ was synthesized on the automated DNA synthesizer by the DMTr ON method. DMTr-(PU)$T_2AT_2GT_2$ was deprotected in 28% NH_3aq at 25°C for 16 hours. After removal of ammonia by speed-vac, the solution was passed through a 0.45 μm filter and the solution was analyzed by reversed-phase HPLC with a linear gradient over 20 min from 0% to 50% CH_3CN in 50 mM AF. The major product was purified, desalinated by reversed-phase HPLC, and identified by MALDI TOF mass spectrometry: DMTr-(PU)$T_2AT_2GT_2$, calcd for $C_{118}H_{136}N_{24}O_{60}P_8$ 3099.3 $[M-H]^-$; found 3097.7. The degradation of the DMTr-containing moiety of DMTr-(PU)$T_2AT_2GT_2$ was carried out by heating in 50% ethylenediamine aqueous solution at 70°C for 8 hours. The solution was neutralized by AcOH. The solution was passed through a 0.45 μm filter and the solution was analyzed by reversed-phase HPLC. The major product was purified, desalinated by reversed-phase HPLC, and identified by MALDI TOF mass spectrometry: p$T_2AT_2GT_2$, calcd for $C_{80}H_{103}N_{22}O_{54}P_8$ 2484.6 $[M-H]^-$; found 2484.6.
